# Interpretable machine learning models for beta thalassemia prediction: an explainable AI approach for smart healthcare 5.0

**DOI:** 10.3389/fmed.2025.1688645

**Published:** 2026-01-14

**Authors:** Maria Abbas, Muhammad Bilal Shoaib Khan, Abdul Hannan Khan, Anas Bilal, Asaad Algarni, Raheem Sarwar

**Affiliations:** 1 Department of Computer Science, Green International University, Lahore, Pakistan; 2 College of Information Science and Technology, Hainan Normal University, Haikou, China; 3 Department of Computer Sciences, Faculty of Computing and Information Technology, Northern Border University, Rafha, Saudi Arabia; 4 Operations, Technology, Events and Hospitality Management (OTEHM), Manchester Metropolitan University, Manchester, United Kingdom

**Keywords:** beta thalassemia carrier (BTC), explainable AI (XAI), neural networks, recurrent neural networks, long short term memory

## Abstract

**Introduction:**

An inherited blood disorder that bounds the production of beta globin, an important protein that has a handsome contribution in the development of hemoglobin and Red Blood Cells (RBC). This protein also enables cells to carry oxygen to tissues throughout the human body. Genetic variation in hemoglobin beta gene signals the body to make beta globin chains is the cause of beta thelasemia with three major types major, intermediate and minor. There is a need of an expert system for the diagnosis of this particular disease.

**Methods:**

This study introduces an interpretable Expert system for the prediction of Beta Thelesemia incorporating Explainable AI (XAI) techniques to enhance clinical needs. Principle component Analysis (PCA) with Synthetic Minority Over-sampling Technique (SMOTE) is applied on the Beta Thalassemia Carrier (BTC) dataset 5066 patients to reduce the dimentiality and balance the output classes. Machime learning classifiers such as Neural Networks, Recurrent Neural Networks and Long Short Term Memory (LSTM) is applied.

**Results:**

The latest one will give the 99.30% accuracy, 99.33% precision, 99.33% recall, 99.33% specificity, and 99.33% f1 score.

**Discussion:**

Furthermore ensuring the models transparency and interpretability, the proposed method integrates SHapley Ad-ditive exPlanations (SHAP) and Local Interpretable Model-Agnostic Explanations (LIME), enabling both global and local interpretability of model predictions. SHAP gives us insight into important features at the global level, while LIME explains individual predictions, making the model's decisions more comprehensible for clinical applications.

## 1 Introduction

Thalassemia describes a class of genetic hemoglobin disorders characterized by deficient synthesis of several globin chains, which affect hemoglobin production and induce persistent hemolytic anemia. The illness primarily falls into two main categories based on the defective globin chain: alpha thalassemia and beta thalassemia. Alpha thalassemia is produced through abnormalities or deletions in the HBA1 and HBA2 genes, which generate alpha globin chains, while beta thalassemia is developed by small changes in the gene known as HBB, which codes the sequence of beta globin chains in hemoglobin. The acuteness of the disease is based on the total number and nature of defective genes. Beta thalassemia is the most clinically important form and often requires long-term medical treatment. These are three different clinical categories of beta thalassemia as major, intermediate, and minor. Each of these categories differs according to the level of severity of anemia, dependence on transfusion, and overall medical attention. Beta Thalassemia major, the most serious kind, typically develops in early childhood and needs regular blood donations as well as iron chelation treatment, posing major healthcare and financial difficulties (1). It is particularly prevalent among people from the Middle East, the Mediterranean region, and Southeast Asia. Based on the World Health Organization (WHO) report, around 1.5% of the world population is affected by beta thalassemia, with roughly 60,000–100,000 new cases diagnosed each year (2, 3). This underlines the significance of identifying, diagnosing, and managing the disease (4).

Several tests are needed to differentiate between iron deficiency anemia and beta thalassemia. These tests comprise liquid chromatography with high performance, serum iron straight, full blood count, ferric, and HBA2, and iron binding ability. However, such tests are costly and not usually accessible in resource-limited settings (5). Although these conventional methods remain the diagnostic standard, their dependence on specialized equipment and expertise often delays early or carrier detection. This limitation highlights a clinical gap that motivates the adoption of computational and machine learning–based tools capable of providing faster, cost-effective, and reliable screening support to complement existing diagnostic practices.

In numerous domains of research, machine learning (ML) methods have demonstrated remarkable success in delivering accurate and meaningful outcomes. Their applications in healthcare are particularly transformative, enabling intelligent oversight, improved diagnostics, and cost-effective disease management. For example, computer-based systems have evolved to diagnose conditions such as beta-thalassemia with enhanced precision and lower operational costs. The effectiveness of machine learning (ML) models varies across domains, as shown by recent advances in AI-driven disease prediction and precision diagnostics (6, 7). ML has proven effective in analyzing electronic health records and detecting maternal complications during SARS-CoV-2 infections (8). It has also contributed to understanding inflammatory and cardiovascular disorders through molecular insights such as SIRT6-mediated inflammation and Tyk2/STAT1 pathway regulation (9, 10). Furthermore, hybrid and ensemble learning models have enhanced drug–target interaction prediction and biosensing applications (11–13). In genetic studies, molecular characterization has identified common β-thalassemia mutations, emphasizing the value of early AI-assisted screening (14). More recently, explainable and graph-based AI models have shown promise in medical imaging and disease–gene prediction (15, 16), underscoring ML's growing role in next-generation healthcare innovation.

Additionally, studies incorporating fluid–solid coupling in hemodynamic analysis further highlight ML's potential in computational medicine (17). Several algorithms, including fuzzy logic (18), deep neural networks (19, 20), K-nearest neighbors (21, 22), support vector machines (23), and deep extreme machine learning (24), have been successfully employed for the assessment of various medical conditions such as lung disorders (25), kidney diseases (26), brain tumors (27), and iron deficiency anemia (28, 29). Furthermore, deep learning-based models for diabetic retinopathy detection continue to demonstrate the power of AI in improving diagnostic accuracy and clinical decision-making (30–32). Artificial intelligence (AI) has notably advanced the identification and management of hematological and genetic disorders. In hematology, AI-based techniques enhance the study of blood smears, enabling fast and accurate diagnosis of conditions like anemia and leukemia (33). Same as, in clinical genetics, it eases the explanation of genomic data, helping in the diagnosis of rare genetic diseases and individual treatment strategies (34). Moreover, its integration into medical diagnostics has smoothed the process and improved diagnostic results across various domains (35). AI comprises many different areas, including machine learning and deep learning are an essential part in preventive medicine (36). Machine Learning has enormous potential to enhance medical results through utilizing huge amounts of data from patients to develop a computational predictive model and specific treatment plans. Highly complex data, like medical imaging, genomes, and electronic medical records, can be examined by employing machine learning techniques to recognize structure and anticipate results (37, 38). With the possible benefits of machine learning in the healthcare sector, there are problems with data security and morality that still need to be addressed (39). A couple of investigators have previously predicted beta thalassemia utilizing machine learning.

The present research supports the early identification of beta thalassemia through the use of several kinds of machine learning algorithms, like Neural Network (NN), Recurrent Neural Network (RNN), and Long Short-Term Memory (LSTM). Such models are utilized for assessing hematologic and medical information to get acquainted with patterns related to the disease prevalence. Since RNN and LSTM are capable of processing sequential data, these algorithms can be utilized to better understand the intricate connection of the dataset. The research compares the efficiency of multiple ML algorithms with the goal of determining which method is more beneficial for quick and precise diagnosis, eventually helping healthcare professionals with early detection and providing better care for the patients. The following are the primary contributions of the research:

- Proposed a beta thalassemia detection model based on an interpreted LSTM, which employs Explainable AI (XAI) to make transparent predictions.
- Demonstrate that the LSTM model performs better in accurate classification for beta thalassemia detection than other models, NN and RNN.
- An extensive data preprocessing workflow encompassing feature scaling, normalization, encoding, outliers removal, and SMOTE has been put in action to enhance the accuracy of the models.
- Used sophisticated data visualization approaches like heatmap, violin plots, histograms, and bar charts to identify important trends inside the dataset.
- Presented assessments of models and findings efficiently utilizing confusion matrices, line charts, bar charts, and ROC curves to help clinical interpretation.

## 2 Literature review

The most effective method for determining whether a patient has β-thalassemia is through the integration of medical expertise with patient data analytics. However, manual identification remains a challenging task that often leads to diagnostic inaccuracies and difficulties in disease control. To prevent transmission to future generations, developing an automated prediction system for thalassemia carrier detection has become essential. This need is even more critical in developing countries, where the high treatment costs make early detection and prevention strategies more feasible than post-diagnosis management. Addressing this concern, Sadiq et al. (40) proposed a voting-based classifier, SGR-VC, which integrates Support Vector Machine (SVM), Random Forest (RF), and Gradient Boosting Machine (GBM) models to improve the evaluation of β-thalassemia. Using data from the Punjab Thalassemia Prevention Programme, their model achieved an impressive 93% accuracy, outperforming individual classifiers such as SVM, GBM, and RF, thereby underscoring the importance of early and data-driven diagnosis. In Devanath et al. (41) the authors employs machine learning techniques to accurately predict thalassemia as a substitute for conventional approaches such as High Performance Liquid Chromatography. Different ten machine learning techniques underwent the test involving Naïve Bayes, Decision Tree (DT), K-Nearest Neighbors, Extreme Gradient Boost, Logistic Regression, Adaptive Boosting, Support Vector Machine (SVM), Random Forest, Gradient Boosting, and Multilayer Perceptron. Having 100% accuracy, ADA Boosting as the most accurate out of them. In Aswathi et al. (42), the authors presented a sophisticated approach to enhancing the screening of beta thalassemia and sickle cell disease using machine learning techniques to detect carrier status, recognize infectious anemia, and variation from blood test, specifically in the north Indian population. The incorporation of bioinformatics with machine learning increases diagnosis fidelity and scanning effectiveness. In this study (39) focused on the development of machine learning models for the detection of thalassemia, especially in a specific region like southern China where the traditional method can be imprecise and expensive. The study analyzed eight parameters from blood tests, using the data of two hospitals in Shenzhen, employed random forest and support vector machine, achieving an accuracy of 94% for machine learning models and 95% for logistic regression formulas. In Schipper et al. (43), the authors concentrated on refining inherited hemoglobin disorder identification utilizing machine learning techniques such as Extreme Gradient Boosting (XGB) dependent on CBC variables, attaining the highest precision in diagnosing extensive of thalassemia while increasing advanced identification and diagnostic performance. It emphasizes that the XGB model outperformed among ROC of 0.97 for beta-thalassemia, 0.98 for alpha-thalassemia, and 0.95 for discriminating anemia from thalassemia. In Subasinghe et al. (44), the authors discussed the issue of thalassemia, an inherited blood disease common in areas such as the Mediterranean, Middle East, and Southeast Asia, with the objective of developing a framework for the identification of beta thalassemia carriers. Support vector machine (SVM) and probabilistic neural network (PNN) algorithms were employed to evaluate eight blood test results utilizing data from 343 individuals in a Thalassemia Care Center in Sri Lanka. PNN model 2 was most effective in managing class imbalance from oversampling with an accuracy rate of 98.75%. In Fu et al. (45), authors explored the use of a machine learning technique, support vector machine, in Taiwan to discriminate between the anemia of inflammation (AI) and iron deficiency anemia (IDA) using data from 350 patients, which consists of genetic mutation and blood measurement evaluation. SVM model obtained excellent precision compared to extant indices, having an AUC of 0.76 and an average percentage of 0.26, which makes it an effective tool for thalassemia detection in medical applications. In Farooq (46), the authors addressed the problem of beta thalassemia prediction using federated learning (FL) techniques and highlighted how effectively the model recognizes carriers, along with maintaining privacy and safety. It underlines the dire need for a reasonable, quick scanning technique and obtaining 92.38% accuracy. In Haghpanah (47), the authors contrasted three machine learning models such as logistic regression (LR), random forest (RF), and gradient boost model (GBM), utilizing medical data from 624 beta thalassemia major individuals, specifically in the liver and heart to enhance service and monitoring. The models were tested employing the area under the curve, accuracy, specificity, and sensitivity. A maximum AUC of 0.68, along with 75% sensitivity, was identified for cardiovascular overload with iron employing logistic regression. Random forest possessed the greatest accuracy, a specificity of 66%, and a sensitivity of 84% over liver iron overload using an AUC of 0.68. In Ayyildiz and Tuncer (48), the authors confront the issue of discriminating across beta thalassemia and iron deficiency anemia (IDA) utilizing indices of RBC and machine learning techniques such as support vector machine and k-nearest neighbor alongside neighborhood component analysis (NCA) of chosen traits. Numerous indices related to RBC are contained in the dataset, which are analyzed individually for men and women. The outcome reveals excellent efficiency, with an AUC roughly equal to 1, significantly improved performance for both men and women, spanning 94.3%–94.5% and 94.8%–95.5%, accordingly. Although ML-based studies for beta thalassemia classification show promising results, they share common limitations. Most rely on small or single-center datasets and specific populations, often using only red blood cell indices or CBC data, which may limit predictive accuracy. Retrospective data, variable biases, and the need for adjustments across populations or genders further constrain their applicability.

In this article (49) concentrates on a late fusion model that diagnoses beta thalassemia carriers using machine learning methods. Efficient detection and attaining comprehensive 96% accuracy, it utilizes Neural Network, Logistic Regression, Decision Tree, and Naïve Bayes. The late fusion model complication may render it more difficult to apply in the everyday healthcare environment. In Jahan et al. (50), the authors explore machine learning models such as artificial neural networks to identify beta thalassemia trait in antenatal women using red cell indices. Across 15 months, using complete platelet and high-performance liquid chromatography (HPLC), 3,947 patients were tested, and 5.98% of them were diagnosed with beta thalassemia trait. ANN attained 85.95% accuracy, with 83.81% sensitivity and 88.10% specificity, outperforming standalone red cell indices, which had a maximum accuracy of 63.8%. In Kabootarizadeh et al. (51), the authors presented a method that differentiates between iron deficiency anemia and beta thalassemia trait because of their identical lab results. Using complete blood count data of 268 patients in Ahvaz, Iran, the author trained an artificial neural network (ANN) to enhance diagnosis, which obtained the highest accuracy of 92.5% with a specificity of 92.33% and a sensitivity of 93.13%. In Upadhyay (52), the authors address the problem of beta thalassemia, an inherited blood disease common among Mediterranean, African, and Southeast Asian populations (5%–30% carriers). This research will build an artificial neural network (ANN) model for earlier beta thalassemia recognition utilizing quantitative blood test outcomes, assisting the pre-marriage therapy to avoid serious complications. The ANN approach, which employed a feed-forward backpropagation network with 11 hidden neurons, was validated on 39 instances after its training on 100 instances. Owing to model dependency on previous data, which could not accurately represent the wide range of thalassemia cases. In Das et al. (19), authors proposed machine learning approaches including artificial neural network, decision tree, and Naïve Bayes to tackle the issue of scanning for beta thalassemia trait and discriminating carriers from healthier individuals, important for avoiding thalassemia major. It establishes a scoring procedure for BTT classification and the overall evaluation of BTT and HbE features. The beta thalassemia trait detection rate in the PGIMER India dataset remained 100%, yet the specificity was low, 79.25% for BTT and 58.62% for the joint score. In Laengsri et al. (53), the authors presented a web-based tool, ThalPred, to make the distinction between thalassemia trait (TT) and iron deficiency anemia (IDA), two prominent forms of Hypochromic Microcytic anemia in Thailand. The authors developed a machine learning (ML) model utilizing testing results from 186 individuals (40 with IDA and 146 with TT) and assessed five algorithms: Random Forest (RF), K-Nearest Neighbor (KNN), Artificial Neural Network (ANN), Support Vector Machine (SVM), and Decision Tree (DT). SVM classifier employed in ThalPred obtained 95.59% accuracy, AUC of 0.98, and MCC of 0.87 using seven red blood cell features. The tool is freely available, permitting health care providers to acquire scanning outcomes without having a background in machine learning. In AlAgha et al. (54) authors developed a hybrid data mining technique for the classification of beta thalassemia, specifically places such as the Gaza Strip, where typical laboratory tests can incorrectly identify carriers that lack symptoms at all. This research applies different preprocessing techniques like SMOTE and four classifiers, such as Decision Tree, Naïve Bayes, K-Nearest Neighbor, and Multilayer Perceptron, on the dataset collected from Palestine Avenir Foundation to improve carriers' detection. Naïve Bayes predictor achieved an excellent outcome, having 98.81% sensitivity and 99.47% specificity. Despite studies using ANN and other ML algorithms showing promising results, they face common limitations. Most rely on small datasets that may not represent the entire population, affecting generalizability and reliability. Additional challenges include lower accuracy for certain subgroups (e.g., men), potential biases from retrospective data, and insufficient validation across different medical contexts, particularly for distinguishing IDA and BTT.

In (53) the authors explored the beta thalassemia and sickle cell disease, stressing the requirements for reliable and cost-effective test kits in areas with limited resources. It brings focus on the weakness of the classic sickle cells and suggests novel approaches to morphology-based labeling that utilize robotic images and machine learning. The technique effectively recognizes sickle cell disease and beta thalassemia characteristics, obtaining an AUC of about 0.940, a sensitivity of 84.6%, a specificity of 92.3%, and a sensitivity of over 97% for deadly SCD. In this research, Saleem et al. (55) addressed the difficulties of thalassemia detection, which is a blood disorder, causing persistent anemia and complications. Toward enhancing thalassemia prediction using machine learning, it analyzes numerous choices of features and classification. The study explored approaches such as Exploratory Factor Score, Recursive Feature Elimination, Linear Regression, and Chi-Square employing a dataset of 10,322 individuals. Having a success rate of 93.46%, Gradient Boosting Classifier (GBC) excelled all nine predictors that were evaluated. Retrospective biased information and dependency on a few numbers of variables are among the limitations and require more verification over. In Shrestha et al. (56) the authors employed machine learning to categorize people with sickle cell disease (SCD) and beta-thalassemia trait, obtaining a 98.6% sensitivity for SCD recognition and a total sensitivity of 82.4%. In a setting with restricted resources, the enhanced sickling tests exhibited promises of inexpensive and computerized scanning. This study utilized five affordable methods, including automated microscopy and machine learning for categorization. In Jahangiri et al. (57), the authors investigate the use of decision-tree-based techniques to differentiate between iron deficiency anemia (IDA) and beta thalassemia, being the very first use of such models for this identification. The CART, CHAID, E-CHAID, QUEST, CRUISE, and GUIDE techniques were employed to evaluate 144 (aged 18-40) individuals with hypochromic microcytic anemia from Ayat Hospital in Tehran. The main predictor was determined as the Mean Corpuscular Volume (MCV), and CRUISE obtained the greatest precision (AUC = 0.99). The study determines that practitioners may identify IDA and BTT more consistently using a decision tree-based model. In Upadhye and Ram (58), authors investigated the utilization of machine learning (ML) techniques to identify multiple kinds of anemia based on red blood cell (RBC) features to obtain timely diagnosis and to prevent serious medical conditions. Five ML methods were tested to categorize anemia into five groups, such as beta thalassemia trait (BTT), dimorphic anemia (DA), macrocytic blood picture (MBP), microcytic hypochromic anemia (MHA), and normocytic normochromic blood picture (NNBP), utilizing data from 2,000 individual samples that represent no anemia. The methods that exhibited the greatest accuracy, sensitivity, and specificity were the random forest and the decision tree. In Ogino et al. (59), research addressed the problem of recognizing microcytic anemia in kids, especially distinguishing between thalassemia trait and iron deficiency anemia in hospitals and clinics. The authors collected biographical data and blood cell count from 76 children who were sent to a hematological center to perform a retrospective evaluation. A logistic regression framework was developed, utilizing significant variables such as red blood cell indices, average corpuscular hemoglobin concentration, and red blood cell dispersion length. The model revealed robust forecasting performance with a sensitivity of 89.2% and specificity of 92.3% in the first group, and 84.4% sensitivity, 88.9% specificity in the verification group. In Jahan et al. (50) authors explored the utilization of red blood cell counts and machine learning techniques for scanning beta thalassemia trait (BTT) in pregnant women, aiming to determine a less expensive substitute to costly procedures such as HPLC. It concerned 3,947 women, and the diagnosis was determined by complete blood count and HPLC. The research showed that a particular red cell count, like MCHC, was inadequate to perform effective detection, while machine learning models, notably the ANN, worked better with an accuracy of 85.95%. The study determined that gathering red cell count with machine learning, especially ANN, offers an exciting tool; further developments are necessary for improved accuracy and generality over a variety of groups. In Xu et al. (60), research concentrates on developing discriminant functions (DFs) to recognize both microcytic and normocytic TT and investigate thalassemia trait (TT) scanning in the Southern Chinese population. The authors assessed diagnostic abilities in 761 individuals by recognizing the most accurate blood test variable utilizing logistic regression and ROC curve assessment. The recommended DFs exhibited excellent precision with an AUC range of 0.892 (women) and 0.861 (men) for microcytic instances, and 0.857 (women) and 0.969 (men) for normocytic instances. Based on these outcomes, DFs could assist with identifying TT, specifically when conducting epidemiological research. While studies using ML, Chi-square, and other statistical or EQUIST-based methods show promising results, they share several limitations. Most rely on small, single-center datasets with limited feature sets, which may restrict generalizability. Additional issues include retrospective data biases, lack of laboratory confirmation, dependence on specific hematological indices, and insufficient validation across diverse populations and real-world medical settings. All these limitations highlight the need for more generalizable and interpretable models.

Table 1 demonstrates that previous studies on beta thalassemia classification generally rely on small or single-center datasets, which restricts the broader generalization of their results. Most works predominantly apply traditional ML techniques such as SVM, RF, and LR, whereas deep learning-based approaches are still limited in this clinical domain. Moreover, imbalance-handling techniques like SMOTE and explainability strategies (XAI) are rarely addressed, despite their importance for fair prediction and clinical decision support. While our study also utilizes a single dataset, we address several of these gaps by applying deep learning architectures (NN, RNN, and LSTM) and incorporating XAI methods (SHAP and LIME) to enhance the interpretability of the predictive outcomes. This helps bridge the gap between model performance and clinical usability.

**Table 1:** Limitations of the previous work.

**Reference**	**Dataset**	**Techniques**	**SMOT**	**XAI**	**Limitations**
Ogino et al. (59)	76	LR	NA	NA	Small dataset, biases, real-world validation needed
Jahan et al. (50)	3947	ANN, ML on RBC indices	NA	NA	Limited generalizability, RBC indices alone insufficient
Upadhyay (52)	139	ANN	NA	NA	Small dataset, Limited generalizability, No real-world validation
Kabootarizadeh et al. (51)	268	ANN	NA	NA	Small dataset, lab variability, Validation needed for IDA and beta thalassemia trait cases
Laengsri et al. (53)	NA	RF, SVM, LR	NA	NA	Retrospective data bias, limited generalizability
Fu et al. (45)	350	SVM	NA	NA	Small sample size, model needs validation for diverse populations
(?)	NA	Automated microscopy & ML	NA	NA	Limited dataset diversity affecting generalizability
Saleem et al. (55)	10,322	Chi-Square, RFE, LR, GBC	NA	NA	Retrospective data bias, limited features, needs broader validation
Haghpanah (47)	624	RF, GBM, LR	NA	NA	Limited predictors, single-center data
Subasinghe et al. (44)	343	SVM, PNN	NA	NA	Limited dataset, restricted generalizability

## 3 Research methodology

**Figure 1:**
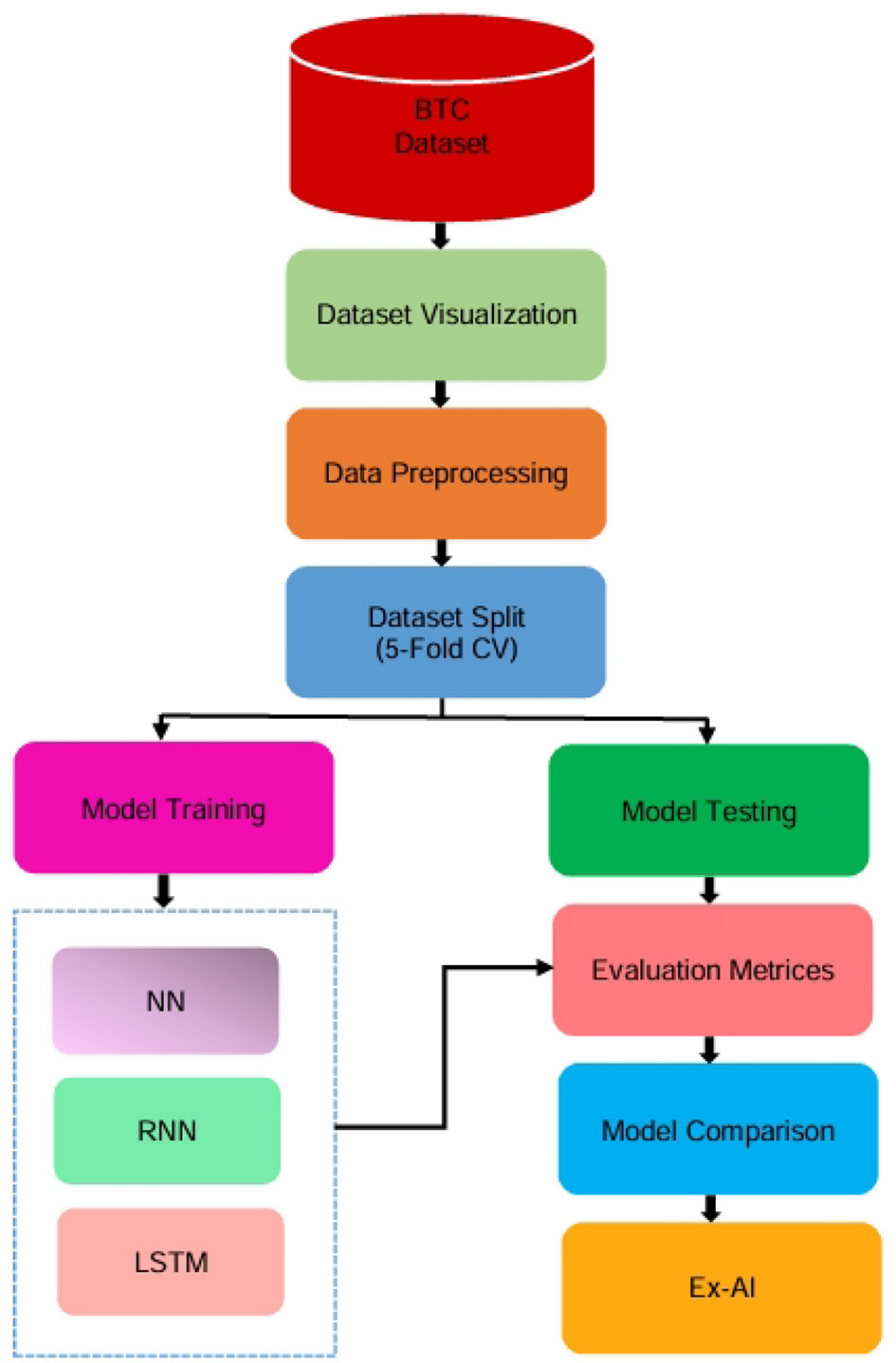
General process flow for beta thalassemia detection.

This section provides a clear overview of the entire research pipeline. A general process flow diagram is presented in Figure 1. This diagram illustrates the sequential steps followed throughout the study—from data acquisition to final model evaluation—ensuring a coherent understanding of the end-to-end methodology.

To address the early and accurate detection of Beta Thalassemia, we adopted a structured machine learning methodology grounded in real-world clinical data sourced from Kaggle. This process involves a careful sequence of steps, from dataset acquisition and preprocessing to dataset splitting, model training, evaluation, and result interpretation. The goal is not only to achieve robust predictive performance but also to enhance transparency in the decision-making process using explainable AI techniques. The complete research workflow is visually summarized in Figure 1, which outlines each phase of the study in detail.

**Figure 2:**
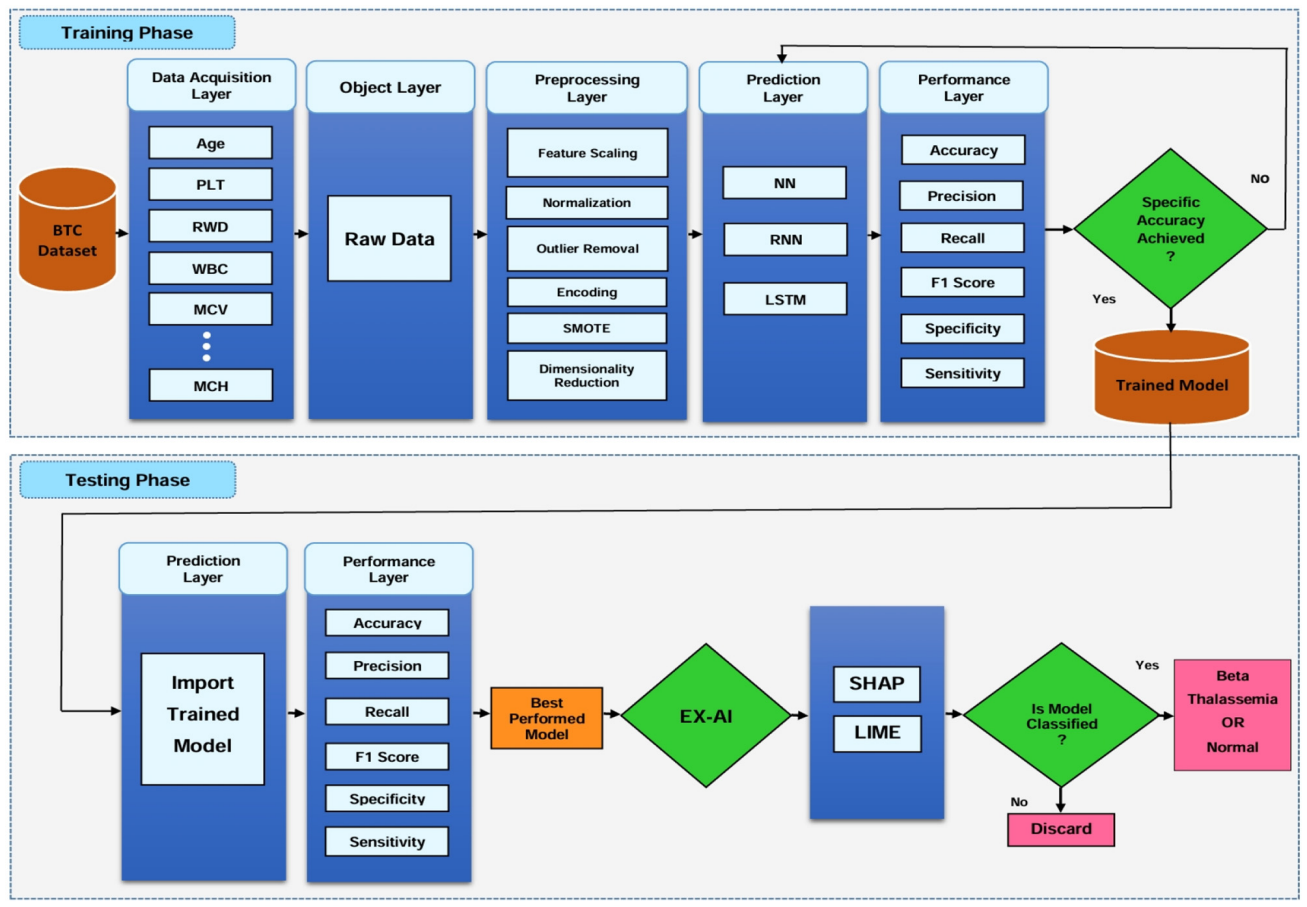
Proposed research methodology diagram for beta thalassemia disease prediction.

While the overall process provides a high-level view, the detailed research methodology shown in Figure 2 elaborates on each component in depth. It outlines specific preprocessing steps, machine learning model integration, and the inclusion of explainable AI techniques such as SHAP and LIME, offering transparency in the prediction process.

### 3.1 Dataset description

**Figure 3:**
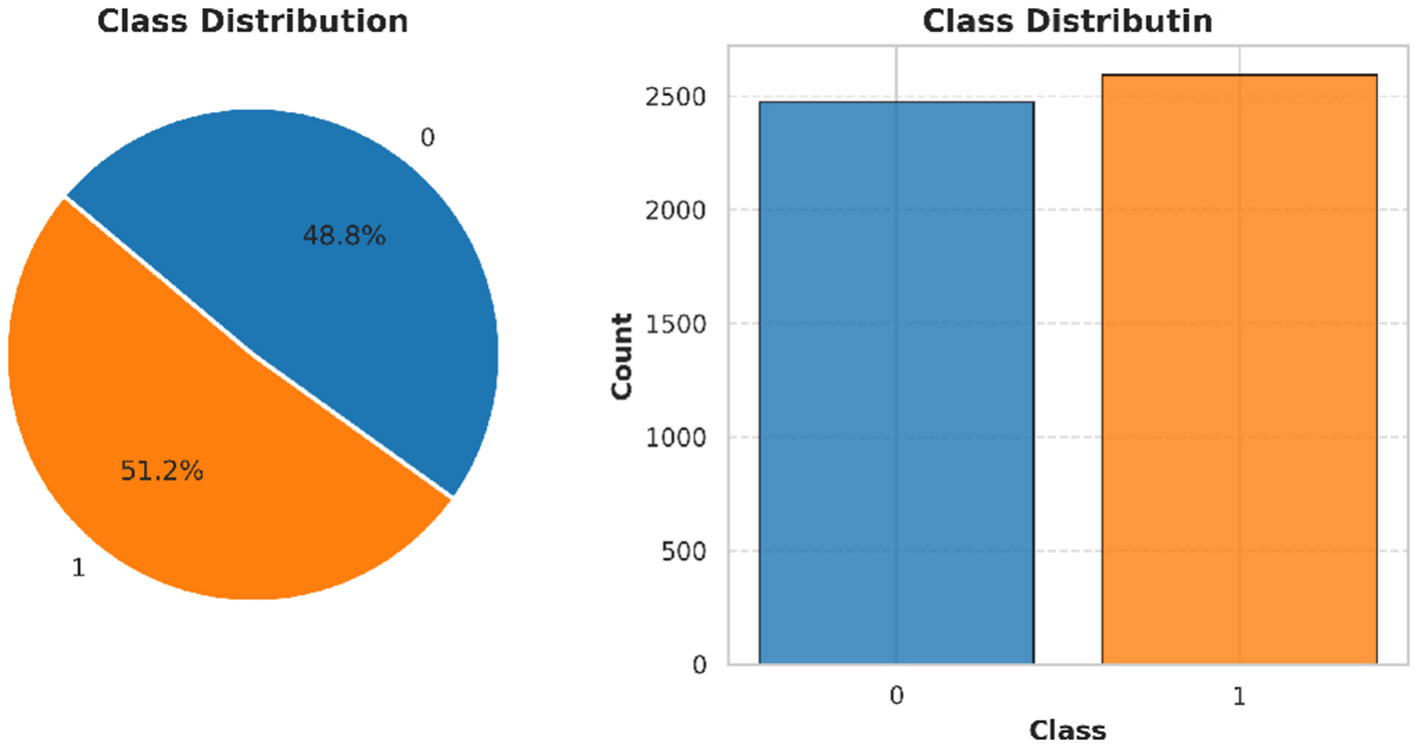
Class distribution of beta thalassemia patients: 51.2% diagnosed and 48.8% undiagnosed cases, highlighting a slight imbalance in the dataset.

**Figure 4:**
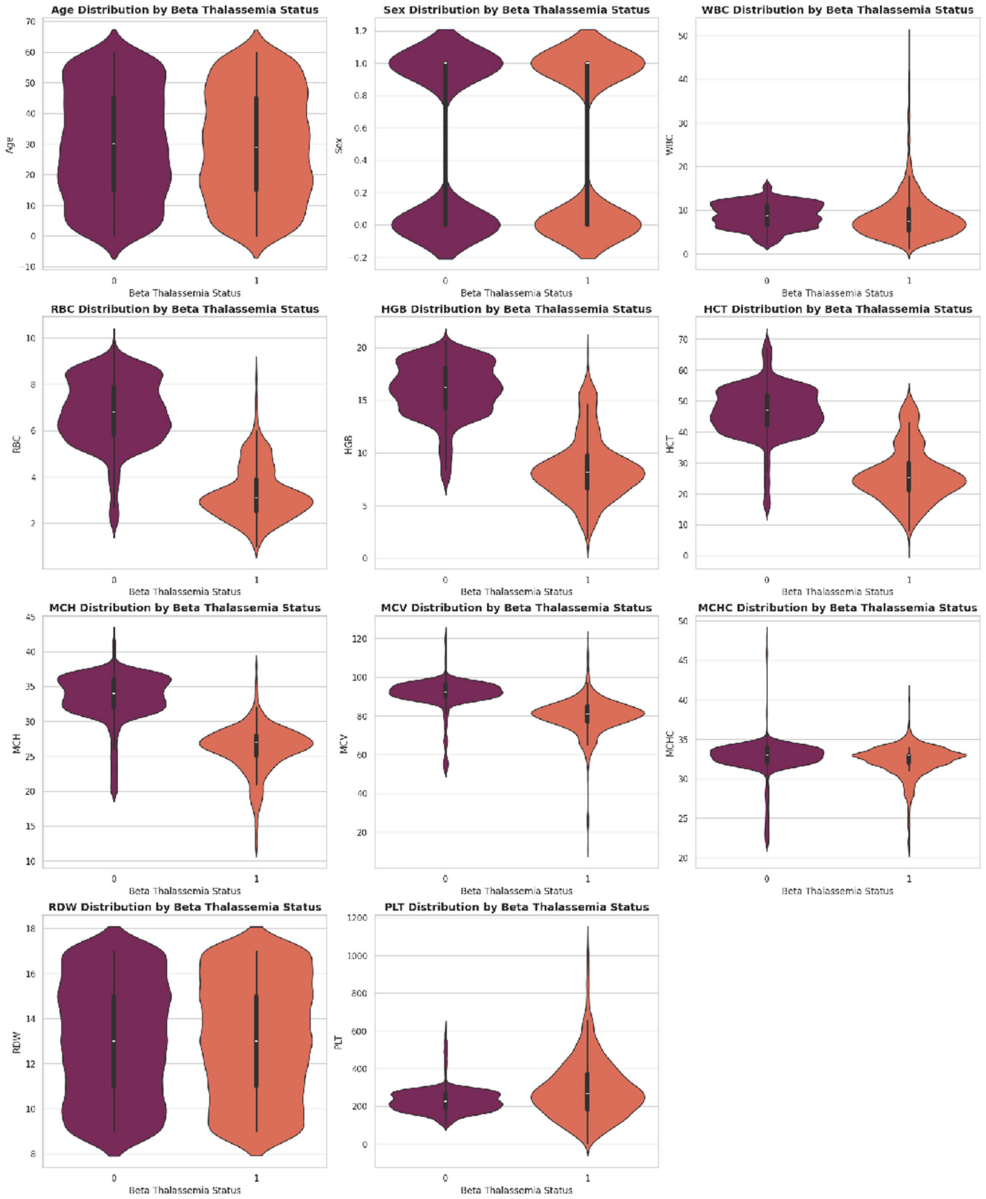
Beta thalassemia status distribution showing the frequency of diagnosed and undiagnosed patients. This visualization provides a clear comparison of class counts in the dataset.

**Figure 5:**
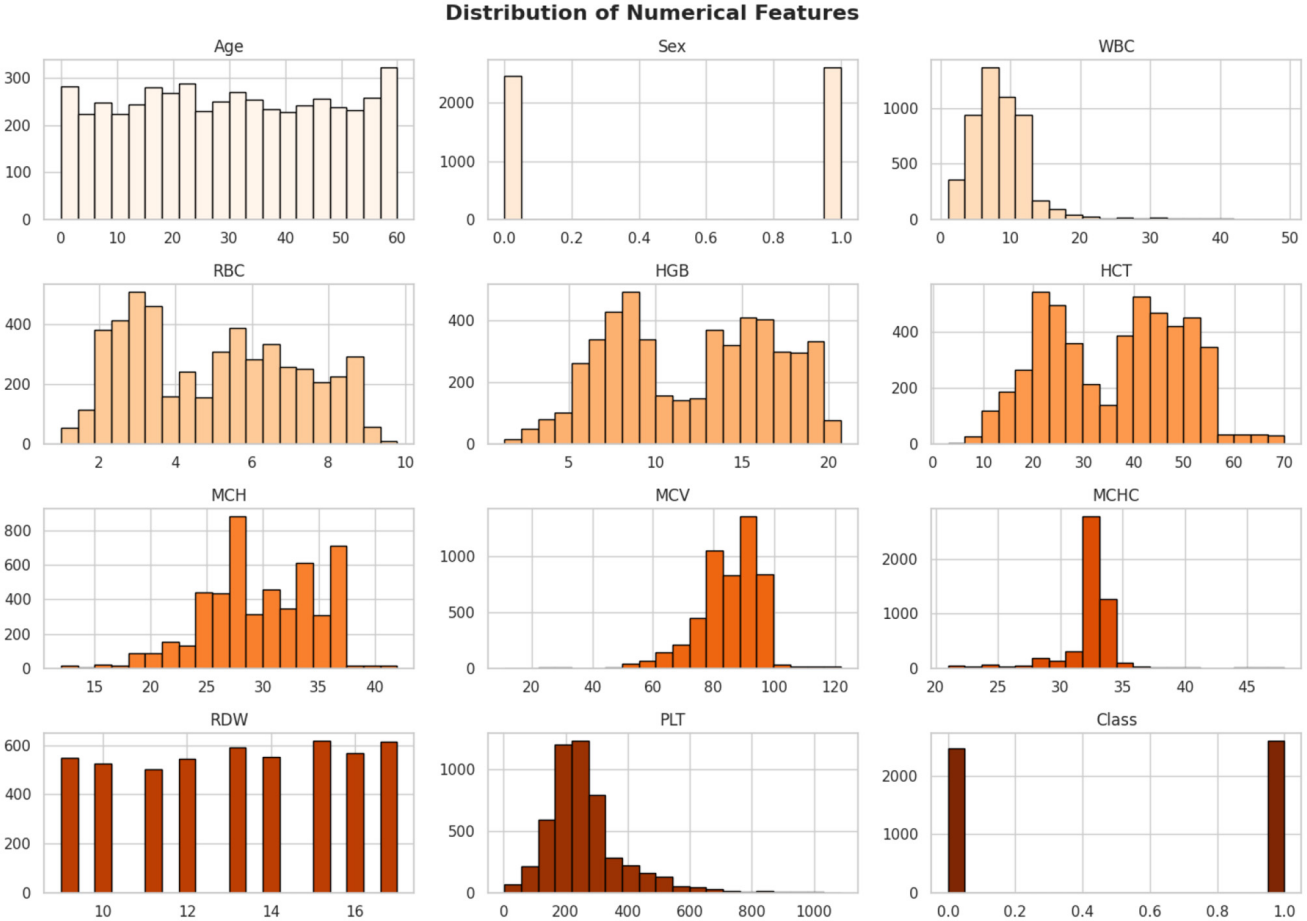
Distribution of key hematological parameters such as Hemoglobin (Hb), MCV, MCH, and RDW. These histograms help visualize data spread, skewness, and concentration trends among patients.

**Figure 6:**
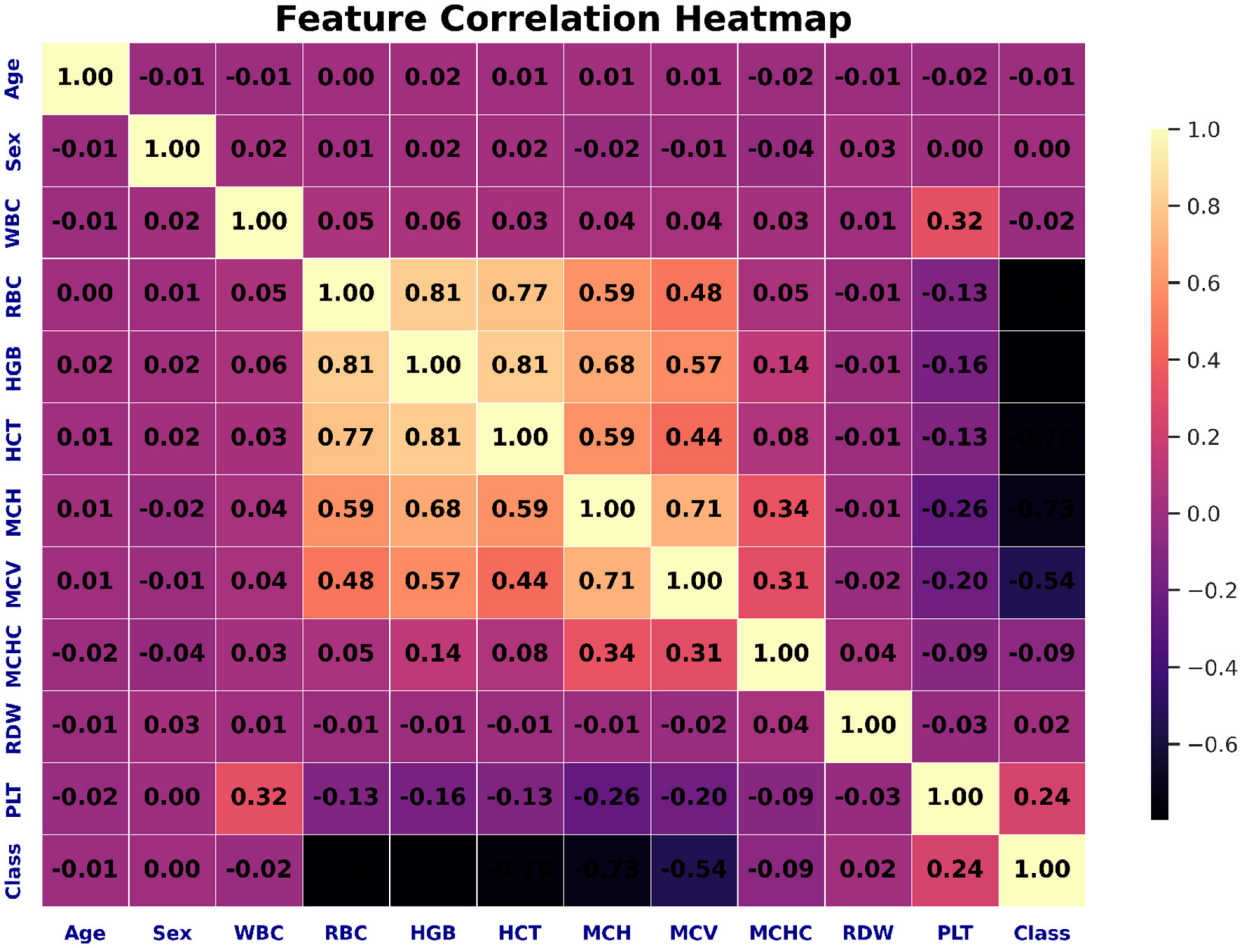
Correlation heatmap illustrating the pairwise relationships among hematological features. Darker shades indicate stronger correlations, helping to identify redundant or highly related variables useful for feature selection.

The dataset is collected from the database of Punjab Thalassemia Prevention Program (PTPP) that comprises 5066 data samples and 12 features for beta thalassemia prediction from the Punjab province of Pakistan (40, 61). The dataset does not have any missing values. The variable “Class” is the classification label (beta thalassemia prediction). The number of patients diagnosed with beta thalassemia is 2,594, and the number of patients not diagnosed with beta thalassemia is 2,472 as further illustrated in Figures 3–6.

### 3.2 Data preprocessing

Data preprocessing is a crucial step in ensuring the quality and reliability of machine learning outcomes, especially in healthcare-related studies. The dataset contained a total of 5,066 samples, which were divided into training 80% (4,052) and testing 20% (1,014), utilizing a stratifying technique to maintain class balance. Testing and training sets are kept independently to strictly avoid data leakage. All preprocessing steps are performed only on training data, and testing data remain unseen during the model training. In this work, we performed the following preprocessing steps on the Beta Thalassemia dataset.

#### 3.2.1 Label encoding

For the classification task, the target variable “Class” is converted into binary format using *label encoding*. The positive class (Beta Thalassemia positive) is encoded as 1, and the negative class (Beta Thalassemia negative) is encoded as 0, making it compatible with binary classification models.

#### 3.2.2 Data cleaning

If no missing values are found, the restoration process is not needed. All attribute values are verified to ensure that they fit inside the expected categories, assuring the data reliability and validity.

#### 3.2.3 SMOT with PCA

**Figure 7:**
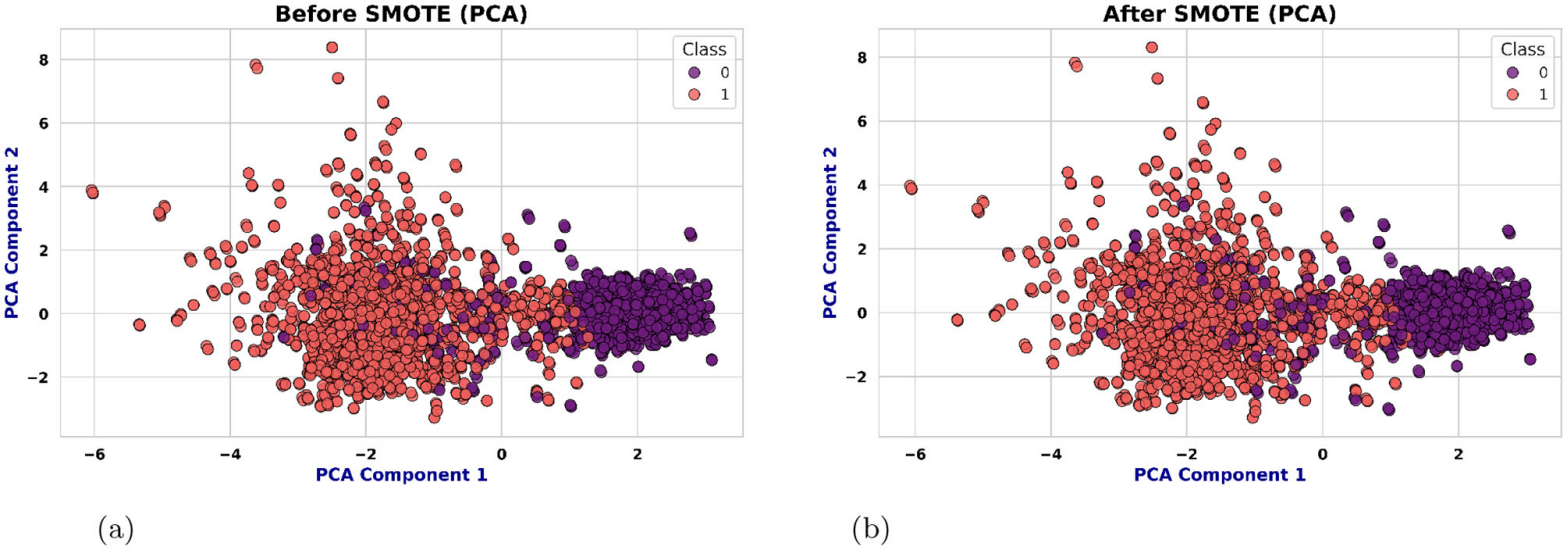
Class distribution pre- and post-SMOTE with PCA. **(a)** Before SMOT: slight class imbalance. **(b)** After SMOT with PCA: balanced distribution.

As shown in Figure 7 the class imbalance is addressed using the Synthetic Minority Over-sampling Technique (SMOTE), which generates synthetic samples for the minority class to ensure balanced learning. Before implementing SMOT, Principal Component Analysis (PCA) is executed for reducing dimensionality. PCA simplifies the data for the purpose of making sure that the synthetic samples are generated in a more organized space of features and reducing noise as well as potential over-fitting.

#### 3.2.4 Normalization and scaling

To improve model performance and convergence, the numerical features are scaled using *Min-Max normalization*, which transforms the feature values into a [0, 1] range. This step ensures that all features contribute equally to the model training process, preventing features with larger ranges from dominating the learning.

### 3.3 Cross validation

**Figure 8:**
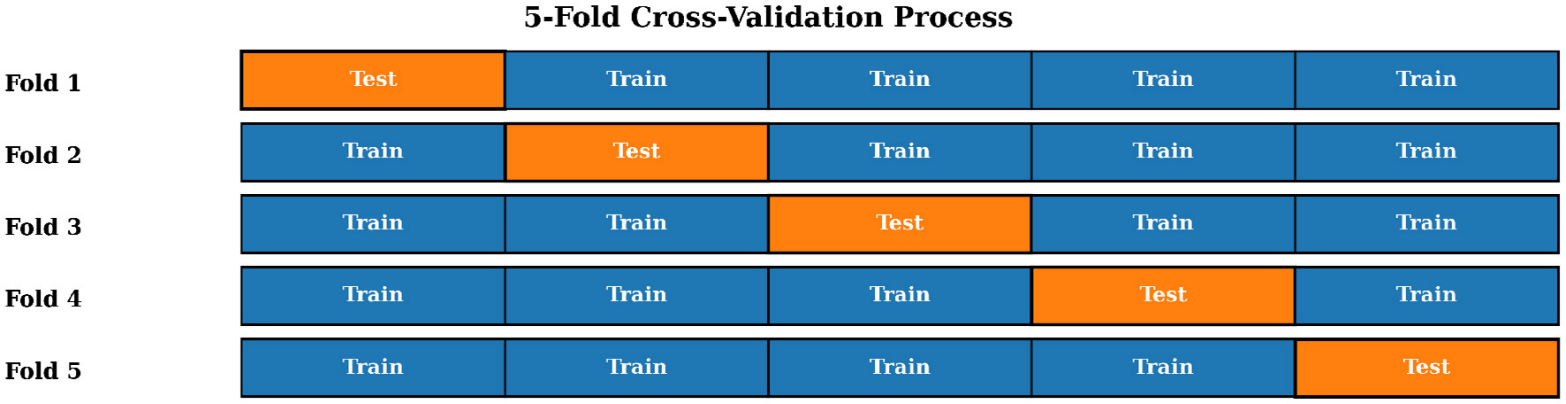
Visual representation of five-fold cross-validation showing the rotation of training and testing subsets across five iterations.

To ensure robustness and generalizability, five-fold cross- validation was employed as presented in Figure 8. The dataset is split into five equal folds, where each fold serves once as a test set while the remaining four serve as the training set. The results across all folds were averaged for final performance reporting.

### 3.4 Model architectures

Investigate the performance of different learning approaches for Beta Thalassemia prediction. Three machine learning models are developed and evaluated: a Feedforward Neural Network (NN), a Recurrent Neural Network (RNN), and a Long Short-Term Memory (LSTM) network. Each model is trained on the same preprocessed and balanced dataset, ensuring a fair comparison.

#### 3.4.1 Neural network

The mathematical model shows how the proposed framework for beta thalassemia disease predictions works. The proposed model consists of three layers input layer, the hidden layer, and the output layer. There's a particular number of neurons in every single layer. There are thirteen neurons in an input layer, five in the hidden layer, and one in the output layer. Applying Gradient Descent Optimization (GDO) requires a couple of stages, like the initial input weight selection, Feed-forwarding GDO of the collections, weight updation in Backpropagation GDO, and bias computing. The activation function utilized by hidden layer neurons is *f*(*x*) = sigmoid (*x*). This function is offered for the input layer in Equation 1, and the sigmoid function of the proposed framework is given in Equation 2. The method's learning element is illustrated by λ

The input layer sigmoid function is depicted as Equation 1.


$$\begin{array}{l} z_{j}=b_{1}+\overset{h}{\underset{k=i}{\sum }}\left(v_{i,j}\times x_{i}\right) \end{array}$$ (1)

The hidden layer sigmoid function is depicted as Equation 2.


$$\begin{array}{l} o_{j}=\frac{1}{1+e^{-z_{j}}};\;j=1,2,3,\ldots ,n \end{array}$$ (2)

The input given by the output layer is shown in Equation 3.


$$\begin{array}{l} z_{q}=b_{2}+\overset{c}{\underset{j=i}{\sum }}\left(w_{j,q}\times o_{j}\right) \end{array}$$ (3)

The output layer activation function is given by Equation 4.


$$\begin{array}{l} o_{q}=\frac{1}{1+e^{-z_{j}}};\;q=1,2,3,\ldots ,r \end{array}$$ (4)

The backpropagation system error is shown in Equation 5.


$$\begin{array}{l} \text{Error}=\frac{1}{2}\underset{q}{\sum }{\left(\sigma_{q}-o_{q}\right)}^{2} \end{array}$$ (5)

The expected output of the system is explained as σ_*q*_. Although, the factual output mentioned is *o*_*q*_.

The rate of change in weight is given in Equation 6


$$\nabla v\propto -\frac{\partial \text{Error}}{\partial v}$$

$$\begin{array}{l} \nabla w_{j,q}=-\epsilon \frac{\partial \text{Error}}{\partial w_{j,q}} \end{array}$$ (6)

The Chain Rule is used in Equation 6 and is represented in Equation 7


$$\begin{array}{l} \nabla w_{j,q}=-\epsilon \frac{\partial \text{Error}}{\partial o_{q}}\times \frac{\partial o_{q}}{\partial z_{q}}\times \frac{\partial z_{q}}{\partial w_{j,q}} \end{array}$$ (7)

If we have


$$\nabla w_{j,q}=\epsilon \left(\sigma_{q}-o_{q}\right)o_{q}\left(1-o_{q}\right)\times o_{j}$$

The impact of putting values in Equation 7 can be represented in Equation 8, which indicates the altered weight.


$$\begin{array}{l} \nabla w_{j,q}=\epsilon \delta_{q}o_{j} \end{array}$$ (8)

Where,


$$\delta_{q}=\left(\sigma_{q}-o_{q}\right)o_{q}\left(1-o_{q}\right)$$

$$\nabla v_{j,q}\propto -\frac{\partial \text{Error}}{\partial o_{q}}\times \frac{\partial o_{q}}{\partial z_{q}}\times \frac{\partial z_{q}}{\partial w_{j,q}}\frac{\partial o_{j}}{\partial z_{j}}\times \frac{\partial z_{j}}{\partial z_{i,j}}$$

$$\nabla v_{j,q}=\epsilon -\frac{\partial \text{Error}}{\partial o_{q}}\times \frac{\partial o_{q}}{\partial z_{q}}\times \frac{\partial z_{q}}{\partial w_{j,q}}\frac{\partial o_{j}}{\partial z_{j}}\times \frac{\partial z_{j}}{\partial z_{i,j}}$$

$$\nabla v_{i,j}=-\epsilon \left[\underset{q}{\sum }\left\{\sigma_{q}-o_{q}\times o_{q}1-o_{q}\times w_{j,q}\right\}\right]\times o_{q}1-o_{q}\times \beta_{i}$$

$$\nabla v_{i,j}=-\epsilon \left[\underset{q}{\sum }\left\{\sigma_{q}-o_{q}\times o_{q}1-o_{q}\times w_{j,q}\right\}\right]\times o_{j}1-o_{j}\times \beta_{i}$$

$$\nabla v_{i,j}=-\epsilon \left[\underset{q}{\sum }\left\{\delta_{q}\times w_{j,q}\right\}\right]\times o_{j}\times 1-o_{j}\times \beta_{i}$$

Where,


$$\begin{array}{l} \nabla v_{i,j}=-\epsilon \delta \times \beta_{i} \end{array}$$ (9)

Such as,


$$\delta_{j}=\left[\underset{q}{\sum }\delta_{q}\times w_{j,q}\right]o_{j}\times 1-o_{j}$$

Weight update across the hidden layer and output layer is expressed in Equation 10, Δ*w*_(_*j, q*) shows the gradient descent with regards to the parameters *w*_*j, q*_


$$\begin{array}{l} w_{j,q}^{+}=w_{j,q}+\mu \text{Δ}w_{j,q} \end{array}$$ (10)

Weight update across the input layer and the hidden layer is shown in Equation 11, Δ*w*_*j*_, *q* describe the gradient descent for the parameters *v*_*i, j*_


$$\begin{array}{l} v_{i,j}^{+}=v_{i,j}+\mu \text{Δ}v_{i,j} \end{array}$$ (11)

μ is the learning rate by which we find the step size to obtain the minimum (local).

#### 3.4.2 Recurrent neural network

In order to maintain information about previous inputs, Recurrent Neural Networks (RNN) maintain a hidden state as in Equation 12.


$$\begin{array}{l} h_{1},h_{2},h_{3},...,h_{T} \end{array}$$ (12)

In the forward pass, the RNN updates its hidden state using Equation 13, which will be written as


$$\begin{array}{l} h_{t}=\sigma \left({W_{x}}_{h}x_{t}+{W_{h}}_{h}{{h_{t}}_{-}}_{1}+b_{h}\right) \end{array}$$ (13)

Where _*W*_*x*_*h*_ is the weight matrix from input to hidden. _*W*_*h*_*h*_ is the recurrent weight matrix from hidden to hidden recurrent. *x*_*t*_ is the input at time step *t*. _*h*_*t*_−_1__ is the recurrent or previous hidden neurons' information.

And *b*_*h*_ is the bias for the hidden layer. σ is typically a non-linear function. Finally, the output at each time step t is given in Equation 14 and will be written as


$$\begin{array}{l} y_{t}=\phi \left({W_{h}}_{y}h_{t}+b_{y}\right) \end{array}$$ (14)

_*W*_*h*_*y*_ is the weight matrix from input to hidden. *h*_*t*_ is the current state of the hidden neuron. *b*_*y*_ is the bias for the output layer and ϕ is the RelU activation function.

#### 3.4.3 Long short term memory

**Figure 9:**
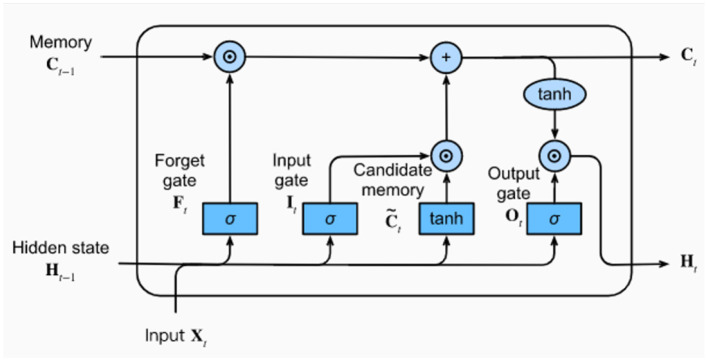
Generic structure of the LSTM.

In LSTM each neuron is called a cell and is composed of input *x*_*t*_, previous information about the hidden state *h*_*t*_−_1_ and previous cell state *C*_*t*_−_1_. Furthermore, the LSTM cell consists of 03 gates and each gate stores the memory component while making it a score after point-wise multiplication using an activation function whose value ranges between 0 and 1. The Information flow will also be regulated using LSTM as presented in Figure 9. The output of the forget gate in the LSTM cell is given in Equation 15 and will be written as


$$\begin{array}{l} f_{t}=\sigma \left({W_{x}}_{h}*\left[h_{t}{-}_{1},x_{t}\right]+b_{f}\right) \end{array}$$ (15)

The output of the input gate of LSTM is written in Equation 16.


$$i_{t}=\sigma (W_{xx}*[x_{t},h_{t}{-}_{1})]+b_{i})$$ (16)

To calculate the hidden state *h*_*t*_, we have to update the cell information as written in Equation 17


$${\tilde{C}}_{t}=tanh(W_{cc}*[h_{t}{-}_{1}),x_{t}]+b_{c})$$ (17)

And the memory cell state Equation 18 will be written as:


$$\begin{array}{l} C_{t}=f_{t}*C_{t}{-}_{1}+i_{t}*{\tilde{C}}_{t} \end{array}$$ (18)

The output gate Equation 19 will be written as:


$$\begin{array}{l} y_{t}=\sigma \left({W_{y}}_{y}*\left[h_{t}{-}_{1},x_{t}\right]+b_{y}\right) \end{array}$$ (19)

Finally, the hidden state will be updated as Equation 20


$$\begin{array}{l} h_{t}=y_{t}*tanh\left(C_{t}\right) \end{array}$$ (20)

### 3.5 Random forest

Along with the deep learning algorithms, a machine learning random forest model was also implemented to provide a conventional ML baseline. This comparison shows the performance gap between DL and ML approaches in beta thalassemia detection.

## 4 Results

The proposed model for the early prediction of beta thalassemia uses machine learning techniques, NN, RNN, LSTM, and RF, using five-fold cross-validation. Model training and validation are implemented in the Google Colab environment using Python. For both training and validation phases, 80% of the dataset is allocated for the training and 20% for testing. The proposed model effectively distinguishes between beta thalassemia and non-beta thalassemia. The classification performance of the proposed approach and individual models is determined utilizing performance metrics including accuracy, precision, recall, specificity, and F1-score, as discussed in the subsequent sections. These metric formulas are written as:


$$\text{Accuracy}=\frac{\text{TP}+\text{TN}}{\text{TP}+\text{TN}+\text{FP}+\text{FN}}$$

$$\text{Precision}=\frac{\text{TP}}{\text{TP}+\text{FP}}$$

$$\text{Recall}=\frac{\text{TP}}{\text{TP}+\text{FN}}$$

$$\text{Specificity}=\frac{\text{TN}}{\text{TN}+\text{FP}}$$

$$\text{F1-Score}=2\times \frac{\text{Precision}\times \text{Recall}}{\text{Precision}+\text{Recall}}$$

The average training and testing accuracy of each model over five folds is presented in Table 2 to highlight baseline performance before visual comparison.

**Table 2:** Fold-wise training and testing accuracy for all models.

**Fold**	**NN train**	**NN test**	**RNN train**	**RNN test**	**LSTM train**	**LSTM test**	**RF train**	**RF test**
Fold 1	80.40	95.50	98.70	97.30	99.80	98.90	92.80	93.20
Fold 2	90.20	95.70	98.80	97.80	99.80	98.60	93.10	92.20
Fold 3	80.90	96.80	99.10	98.40	99.80	99.10	92.90	93.00
Fold 4	78.60	93.80	98.40	98.60	99.80	99.70	92.80	93.40
Fold 5	78.70	96.10	98.60	98.50	99.90	99.60	92.90	92.80
**Average**	**81.80**	**95.60**	**98.70**	**98.10**	**99.80**	**99.20**	**92.90**	**92.90**

We report a 95% confidence interval (CI) to further ensure statistical validity for each performance metric across cross-validation folds in Table 3.

**Table 3:** Performance metrics (mean ± 95% CI) across folds for different models.

**Model**	**Accuracy (%)**	**Precision (%)**	**Recall (%)**	**F1-Score (%)**	**Specificity (%)**
NN	95.56 ± 0.99	95.56 ± 0.99	95.56 ± 0.99	95.56 ± 0.99	95.47 ± 1.79
RNN	98.14 ± 0.48	98.14 ± 0.48	98.14 ± 0.48	98.14 ± 0.48	98.14 ± 0.35
LSTM	99.19 ± 0.40	99.19 ± 0.40	99.19 ± 0.40	99.19 ± 0.40	99.11 ± 0.47
RF	92.91 ± 0.40	92.91 ± 0.40	92.91 ± 0.40	92.91 ± 0.40	91.22 ± 0.81

### 4.1 Statistical significance testing using paired *t*-test

**Figure 10:**
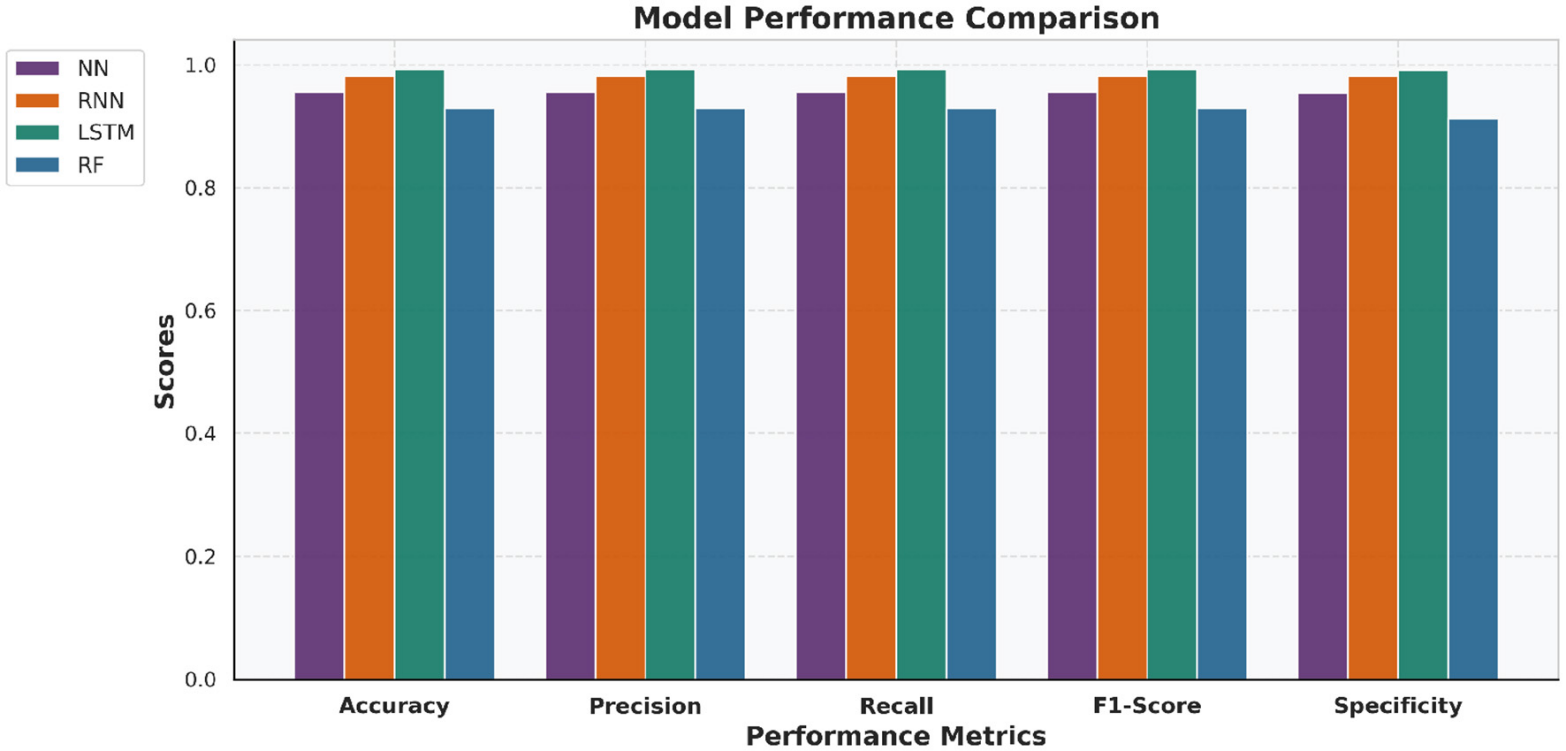
Bar chart comparing NN, RNN, LSTM, and RF performance across key evaluation metrics. The LSTM model consistently achieved superior results across most metrics.

**Figure 11:**
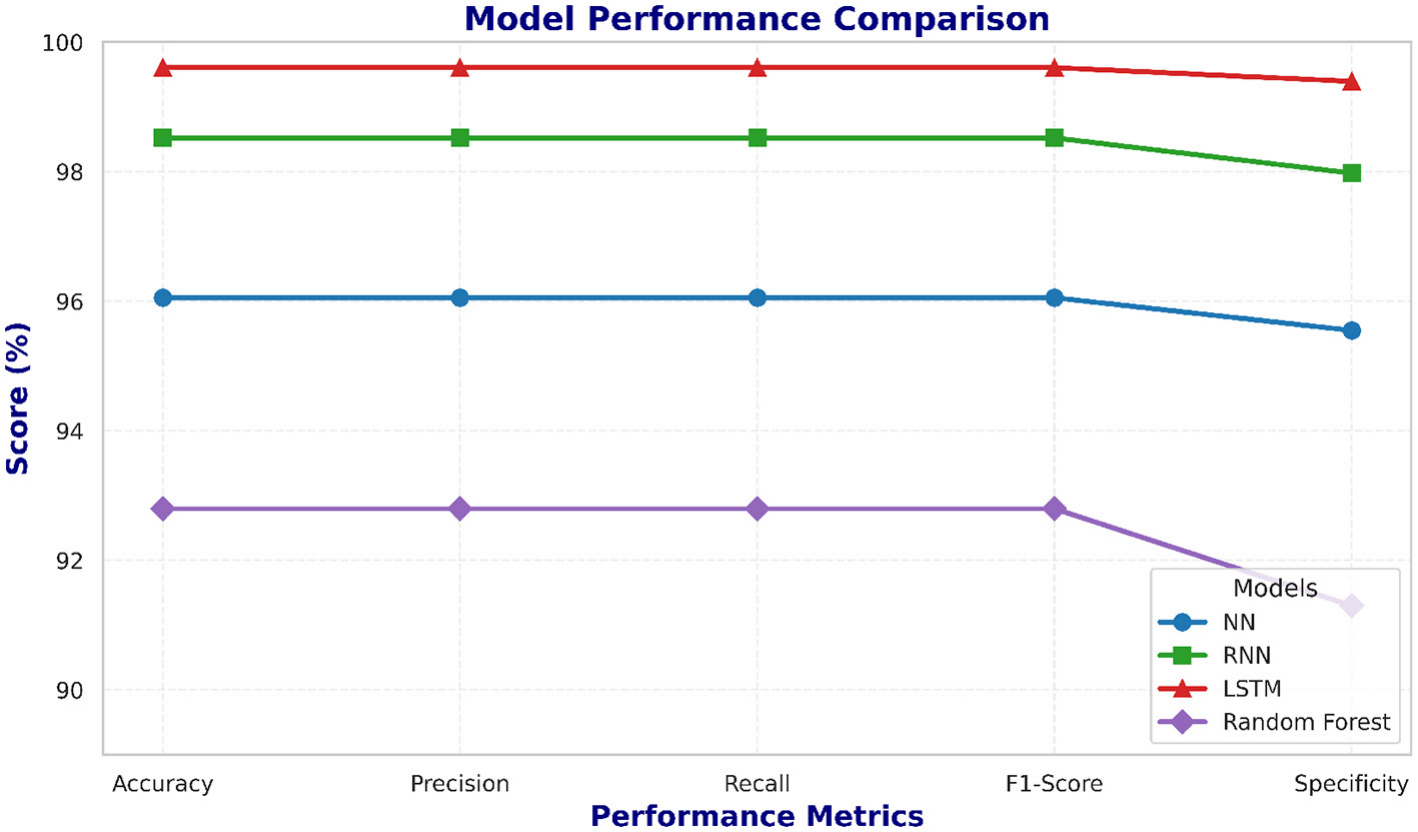
Line chart illustrating trends across evaluation metrics for NN, RNN, LSTM and RF models. The progression highlights LSTM's consistent dominance in predictive performance.

To validate the performance differences among the developed models, a paired *t*-test is applied on the 5-fold cross-validation test accuracies. The statistical results presented in Table 4 show that the LSTM model achieves significantly promising results compared to NN, RNN, and RF models (*p* < 0.05). This statistical observation is also supported in Figures 10 and 11, which provide a bar-wise (Figure 10) and trend-based (Figure 11) comparison across all evaluation metrics, revealing a persistent win for the LSTM model paradigm verifying that the LSTM does out-perform the other four models and this is no random variance in results.

**Table 4:** Paired *t*-test results comparing LSTM with other models.

**Comparison**	***t*-statistic**	***p*-value**	**Significance**
LSTM vs. NN	5.871	0.0042	Significant
LSTM vs. RNN	6.758	0.0025	Significant
LSTM vs. RF	34.671	0.000004	Highly significant

### 4.2 Confusion matrix analysis

Confusion matrices are created to visualize the classification results of each model regarding True Positive (TP), True Negative (TN), False Positive (FP), and False Negative (FN). These matrices offer insights into the predictive behavior of the models and highlight where misclassifications occurred.

- A higher count of TP and TN indicates better classification performance.
- The LSTM model exhibited a lower rate of false negatives, which is crucial in medical diagnosis.

**Figure 12:**
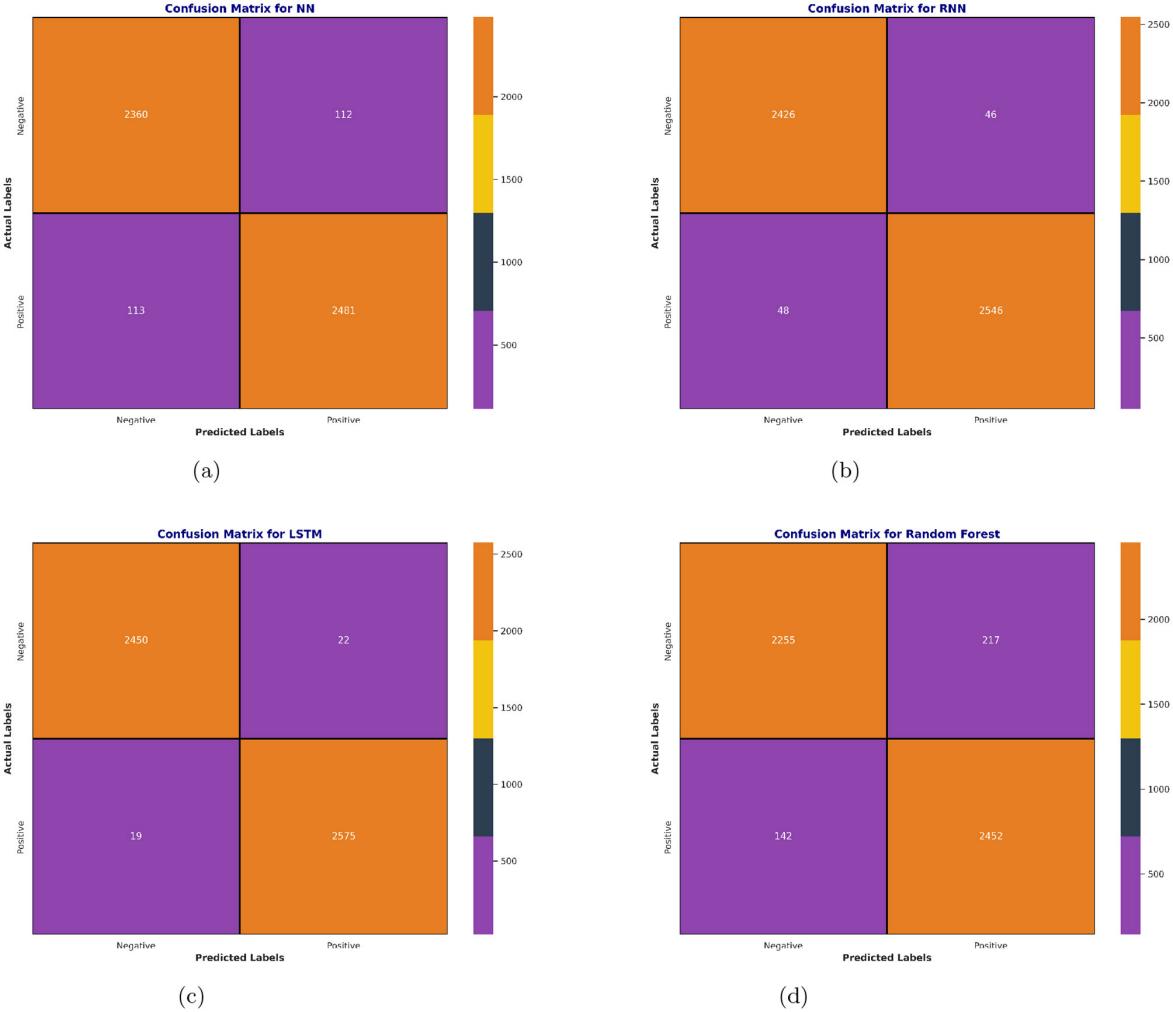
Confusion matrices of all four models. Confusion matrices for **(a)** NN, **(b)** RNN, **(c)** LSTM, and **(d)** Random Forest models, illustrating classification performance across true and false positives/negatives.

Figure 12 presents the confusion matrices for all four models, which provide a more granular view of the true positives, true negatives, false positives and false negatives, yielding a deeper understanding of model-wise prediction accuracy as well as error distribution in the data.

### 4.3 Training and validation performance

The learning behavior of NN, RNN, LSTM, and RF models is evaluated using combined accuracy, loss, and ROC curves. These plots reflect both training and testing performance over epochs as presented in Table 5.

- Accuracy curves indicate consistent learning, with LSTM achieving the highest and most stable validation accuracy.
- Loss curves show smooth convergence, where LSTM recorded the lowest validation loss.
- ROC curves highlight the superior class-separation ability of LSTM, reflected in its higher AUC.

**Table 5:** ROC-AUC and sample ROC points for all models.

**Model**	**ROC-AUC**	**FPR (first 5)**	**TPR (first 5)**
NN	0.9808	[0.0, 0.0, 0.0, 0.001, 0.001]	[0.0, 0.0, 0.003, 0.003, 0.12]
RNN	0.9976	[0.0, 0.0, 0.0, 0.0, 0.0]	[0.0, 0.0, 0.002, 0.002, 0.005]
LSTM	0.9984	[0.0, 0.0, 0.002, 0.002, 0.002]	[0.0, 0.002, 0.674, 0.722, 0.722]
RF	0.9809	[0.0, 0.003, 0.004, 0.004, 0.013]	[0.0, 0.536, 0.63, 0.651, 0.769]

**Figure 13:**
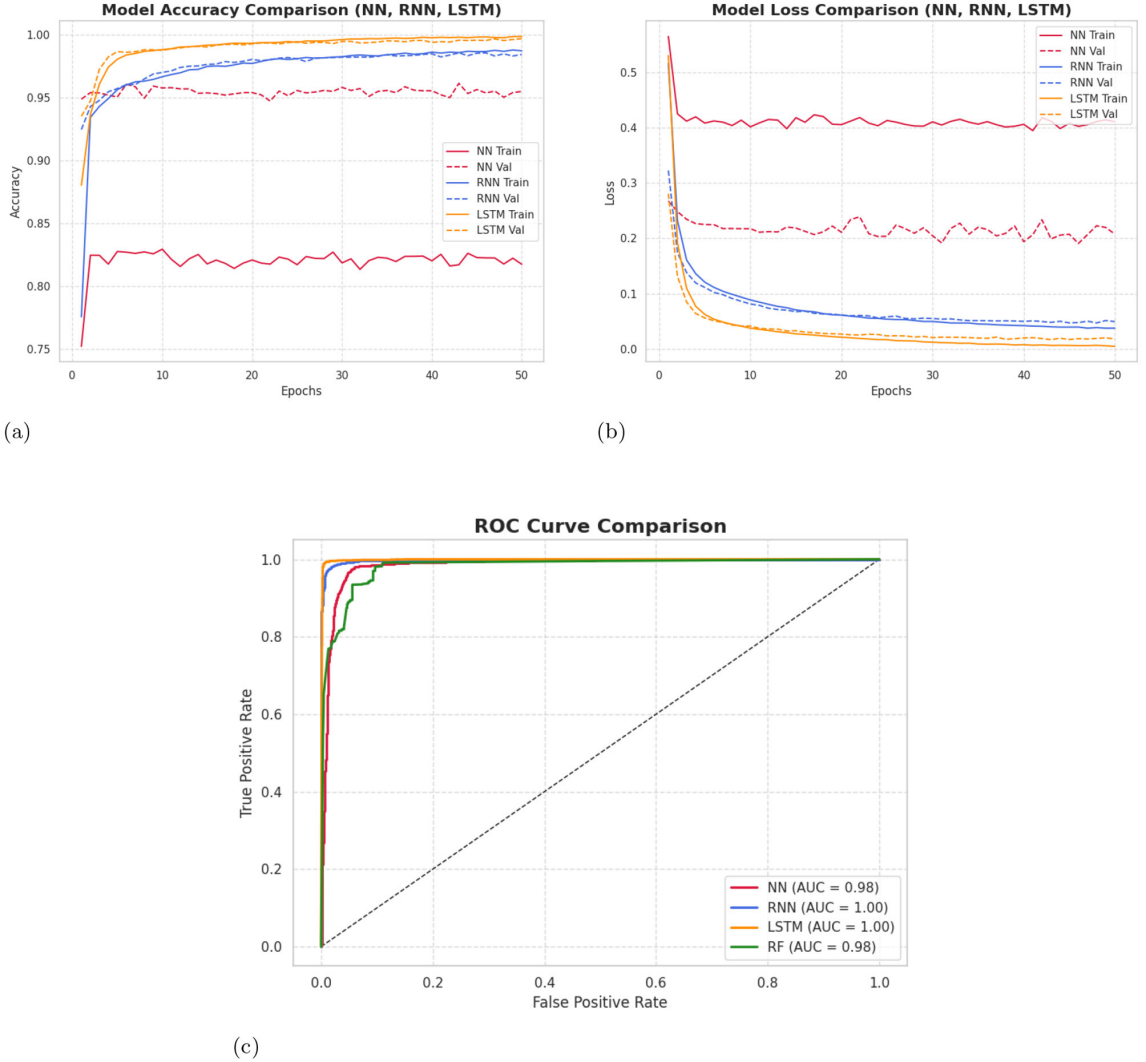
Combined **(a)** accuracy, **(b)** loss, and **(c)** ROC curves for NN, RNN, LSTM and RF models showing both training and testing performance.

Figure 13 shows the combined training and testing accuracy curves, loss curves, and ROC curve for NN, RNN, LSTM, and RF models to illustrate the overall learning behavior and generalization capability and to allow the comprehensive comparison of convergence characteristics and predictive performance across the models.

### 4.4 Explainable predictions using SHAP and LIME for the LSTM and NN model

Explainable Artificial Intelligence (XAI) focuses on creating models that not only deliver accurate predictions but also provide transparent and interpretable reasoning behind those predictions. Model explainability is vital in high-stakes sectors such as healthcare to ensure transparency, confidence, and informed choices. To increase the transparency of the LSTM and NN algorithms' predictions and offer insights into feature significance, SHAP and LIME serve as vital techniques for understanding complicated models.

#### 4.4.1 SHAP feature insights for LSTM and NN

**Figure 14:**
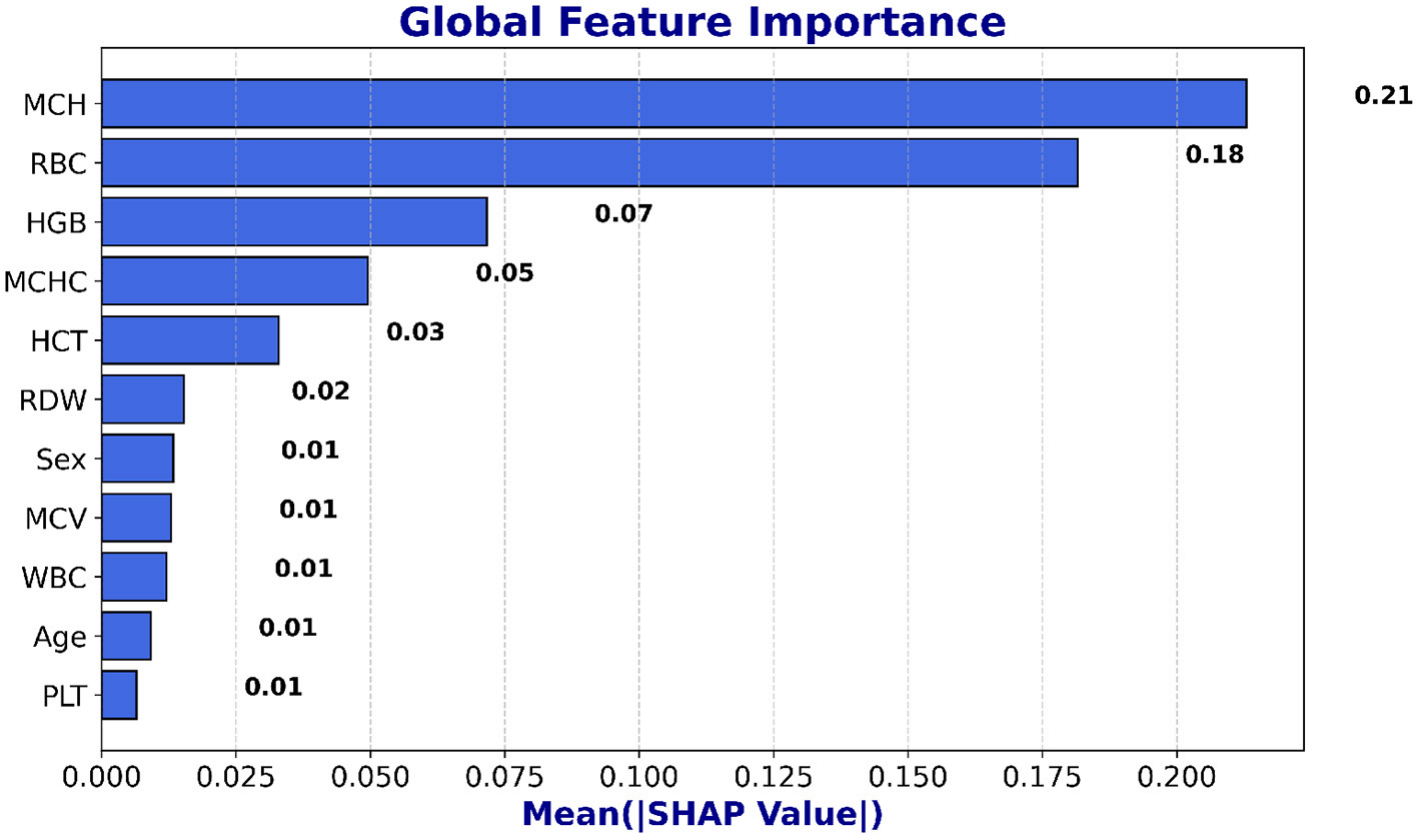
Global interpretability of the LSTM model using SHAP.

**Figure 15:**
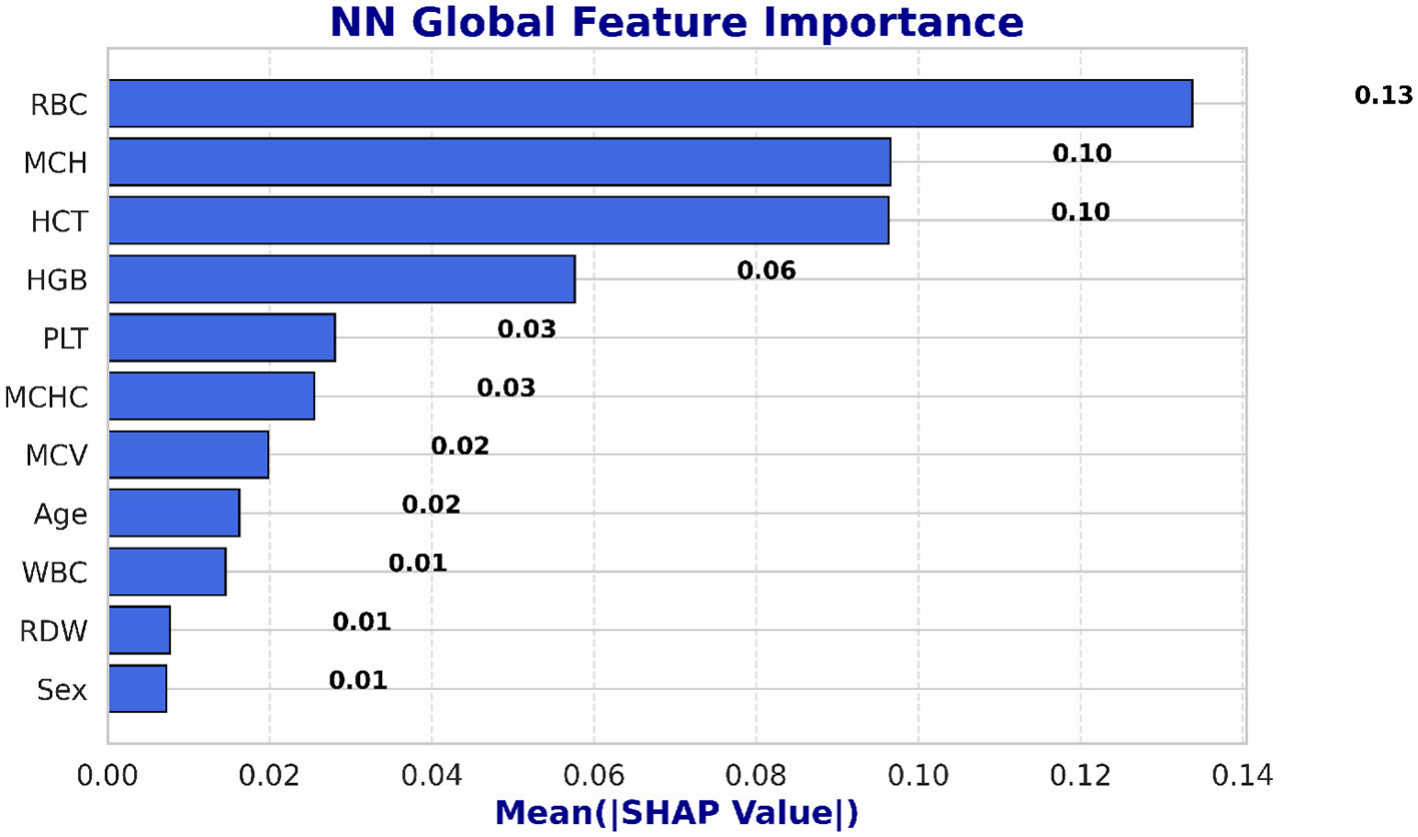
Global interpretability of the NN model using SHAP.

**Figure 16:**
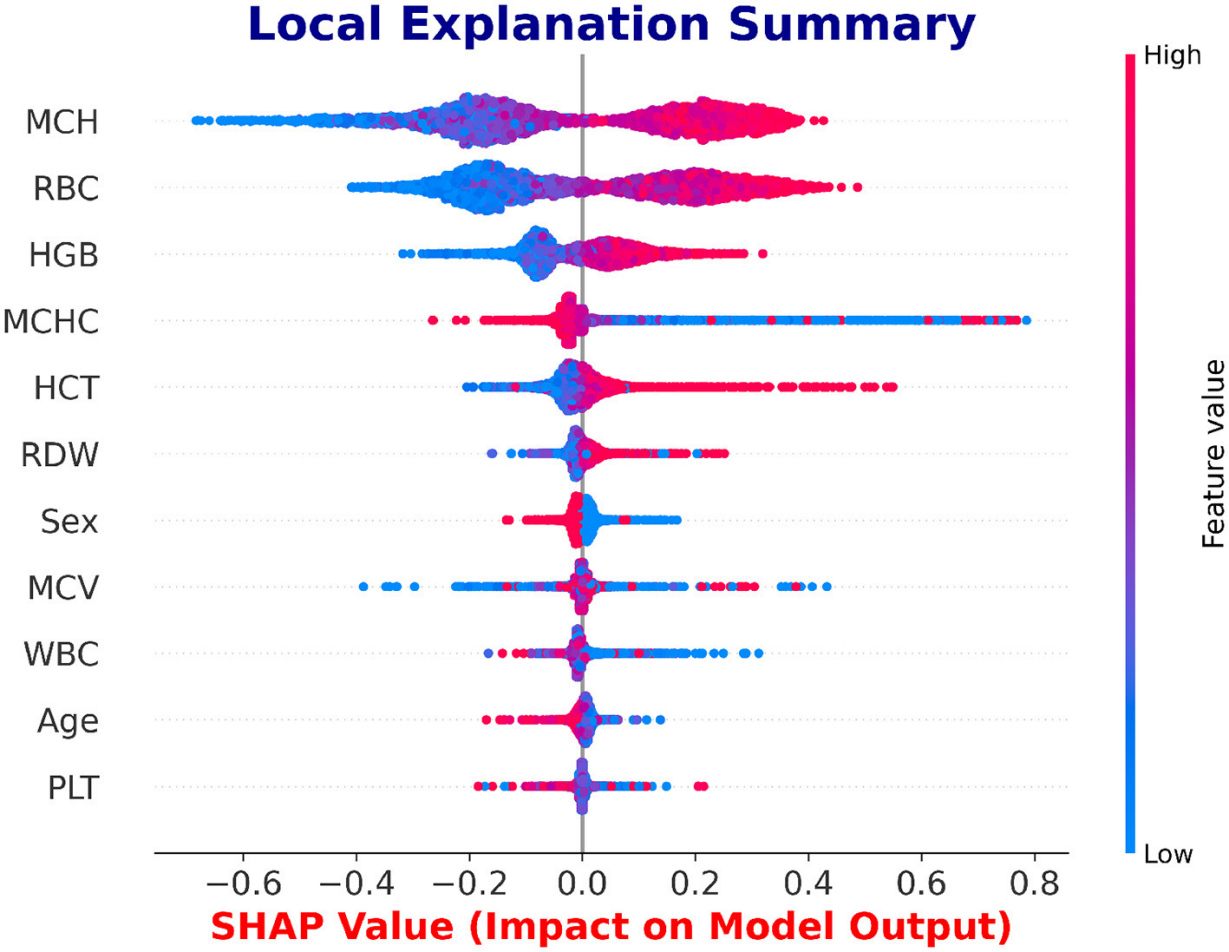
Local interpretability of the LSTM model using SHAP.

**Figure 17:**
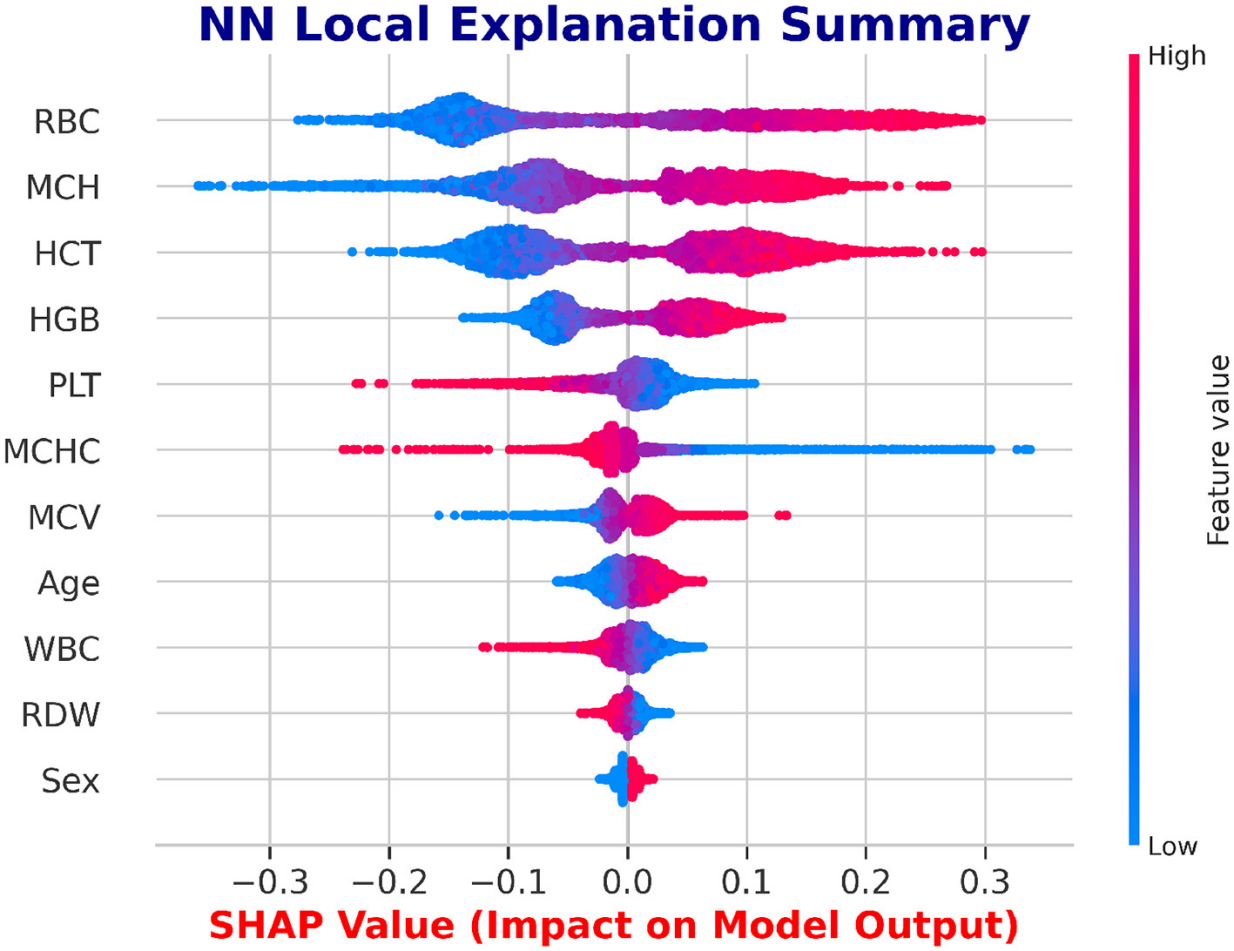
Local interpretability of the NN model using SHAP.

SHAP is utilized to evaluate the global feature significance and local interpretation of the LSTM and NN prediction for the beta thalassemia detection. Three of the most significant features: *Mean Corpuscular Volume (MCV)* is consistently reduced in beta thalassemia, reflecting microcytosis, which has been widely used as a diagnostic marker to differentiate carriers from iron deficiency anemia and other hemoglobinopathies, *Red Blood Cell (RBC)* count is often relatively elevated in beta thalassemia trait despite anemia, since the underlying defect is in globin chain synthesis rather than RBC production, and this distinction is well documented in clinical hematology literature (62), and *Hemoglobin* is significantly decreased in beta thalassemia due to impaired beta globin synthesis, with beta0 thalassemia patients typically exhibiting the most severe reductions compared to beta+ forms (3), which are followed by clinical expertise to predict beta thalassemia are highlighted in the global feature importance plot, which is shown in Figures 14, 15 and feature importance summary in Table 6. Along with global analysis computing SHAP values for an actual patient provide local explanation. The precise contribution of each feature to the model prediction is shown in Figures 16, 17, which display the local explanation detail for a particular patient. These SHAP-based findings increase the LSTM and NN models' clinical utility and understanding, rendering them reliable tools for making medical choices.

**Table 6:** SHAP feature importance summary.

**Feature**	**Mean SHAP value**	**Interpretation/clinical relevance**
MCH	0.21	Most influential; low MCH indicates microcytosis, a key beta-thalassemia marker
RBC	0.18	High importance; reduced RBC count is associated with anemia severity
HGB	0.07	Hemoglobin level; helps distinguish affected vs healthy individuals
MCHC	0.05	Moderate influence; low MCHC may indicate hypochromia
HCT	0.03	Contributes to classification; related to blood volume and red cell proportion
RDW	0.02	Slight effect; reflects variation in red cell size
Sex	0.01	Minimal impact on prediction
MCV	0.01	Low impact; indicates average red cell size
WBC	0.01	Minor influence; primarily used for overall blood profile
Age	0.01	Minimal effect
PLT	0.01	Minor effect on model prediction

#### 4.4.2 LIME feature insights for LSTM and NN

**Figure 18:**
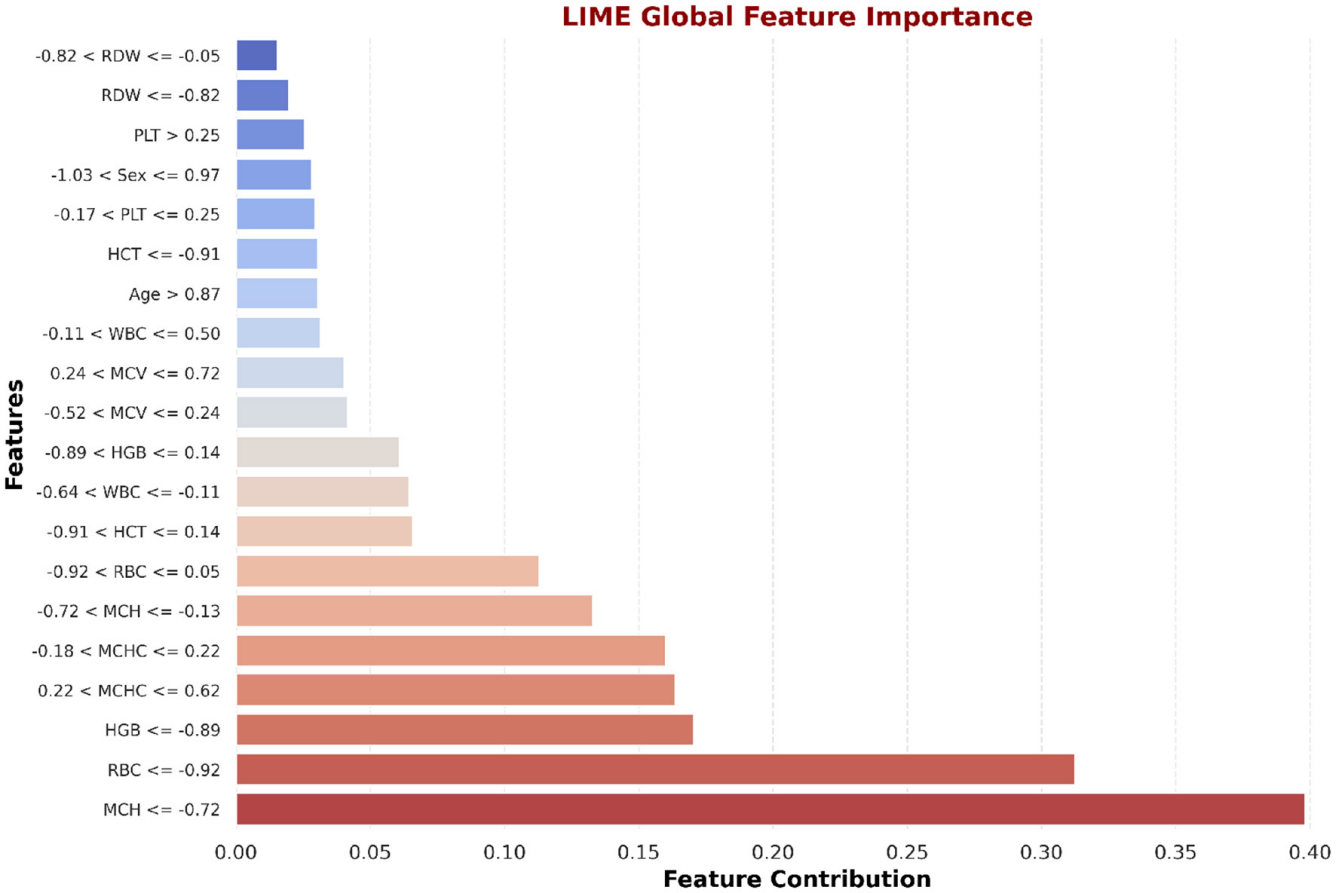
Global interpretability of the LSTM model using LIME.

**Figure 19:**
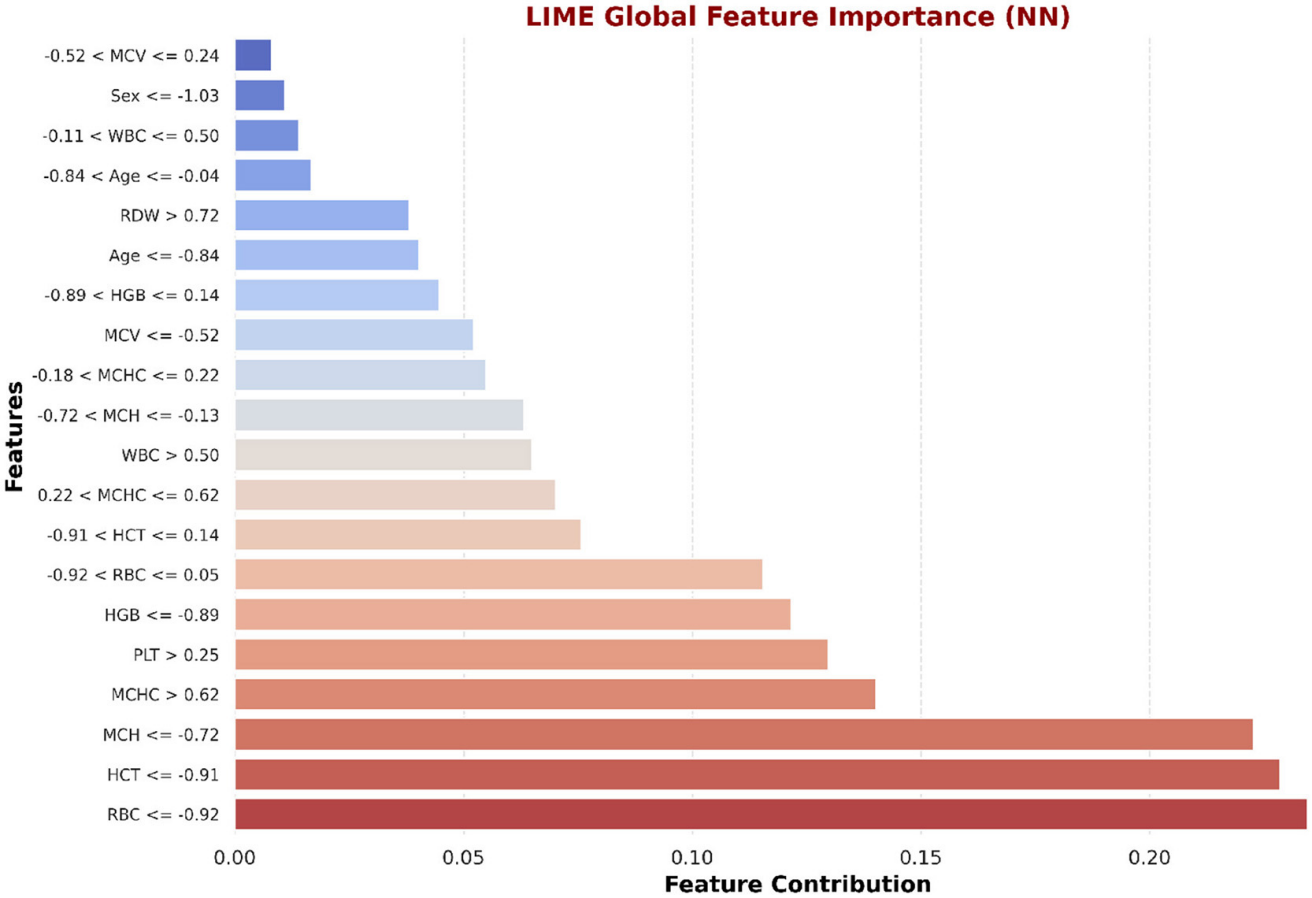
Global interpretability of the NN model using LIME.

**Figure 20:**
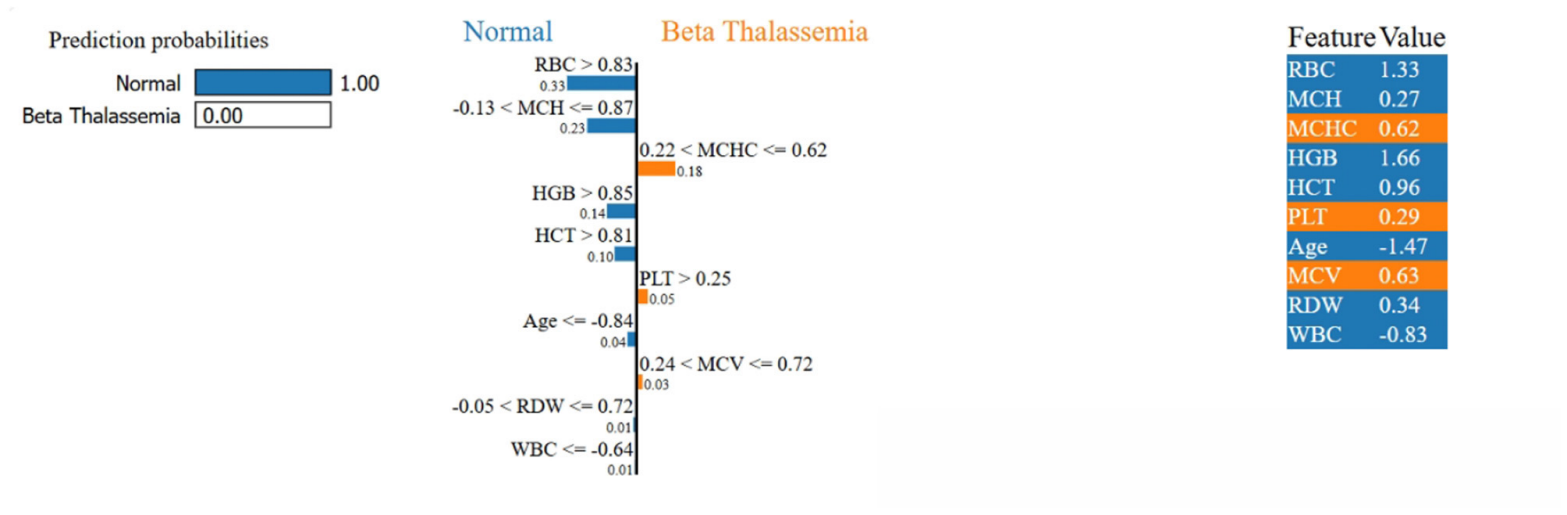
Local interpretability of the LSTM model using LIME.

**Figure 21:**
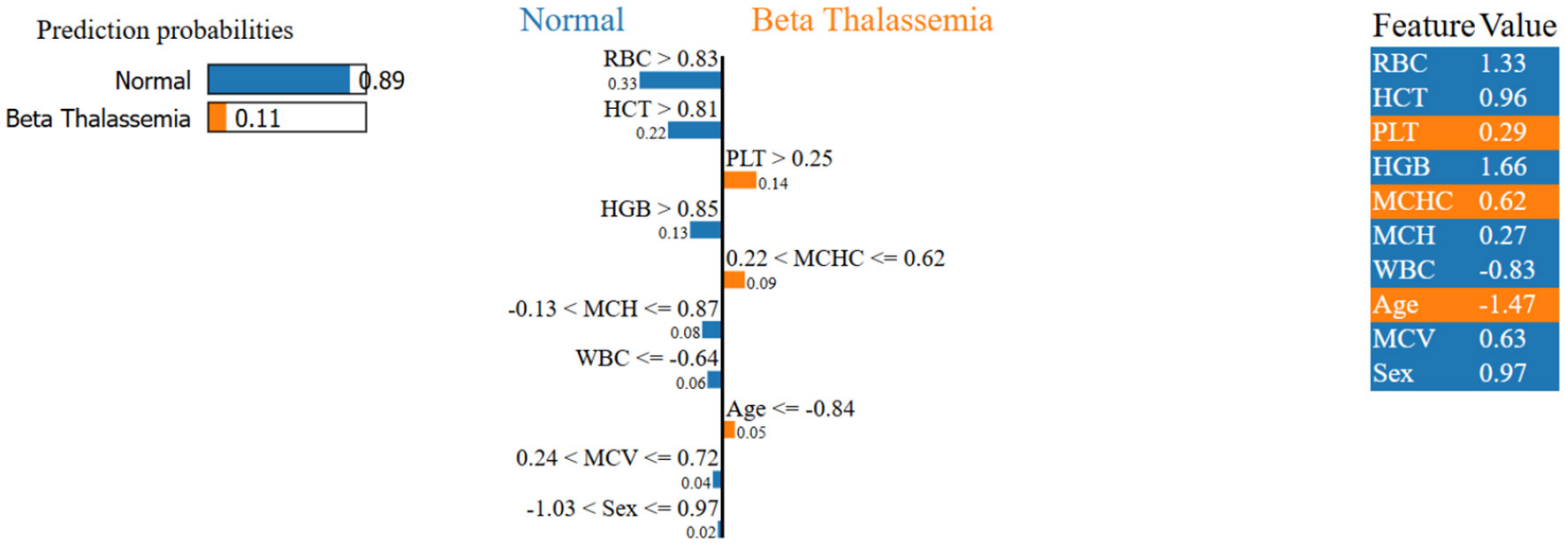
Local interpretability of the NN model using LIME.

LIME is utilized to explain the LSTM and NN model prediction for the identification of beta thalassemia. By employing explainable surrogate models to locally resemble advanced black box models, this approach enables the interpretation of both local prediction and the importance of global features. The consequent plot, as appears in Figures 18, 19, and feature importance summary in Table 7, highlights the features that have the most effect on the predictions made by the model. The most significant features are *Red Blood Cell (RBC)* count (62), *Hemoglobin* (3), and *Mean Corpuscular Volume (MCV)* (62). Furthermore, the local interpretation for a specific case is shown in Figures 20, 21. The model visibility is improved through this specific case clarification, which explains the way each feature contributes to the model prediction for that specific individual.

**Table 7:** LIME feature importance summary.

**Feature**	**Mean LIME contribution**	**Interpretation/clinical relevance**
MCH	0.39	Most influential; low MCH indicates microcytosis, a key beta-thalassemia marker
RBC	0.32	High importance; reduced RBC count is associated with anemia severity
HGB	0.18	Hemoglobin level; helps distinguish affected vs healthy individuals
MCHC	0.16	Moderate influence; low MCHC may indicate hypochromia
RBC (upper bound)	0.11	Supports classification; reflects red blood cell count range
MCH (upper bound)	0.14	Slight effect; indicates variations in MCH contributing to prediction
WBC	0.07	Minor influence; primarily used for overall blood profile
HCT	0.07	Contributes to classification; related to blood volume and red cell proportion
MCV	0.04	Low impact; indicates average red cell size
Age	0.03	Minimal effect
PLT	0.03	Minor effect on model prediction
Sex	0.03	Minimal impact on prediction
RDW	0.02	Slight effect; reflects variation in red cell size

## 5 Discussion

As summarized in Table 8, a comparative analysis with existing beta-thalassemia prediction studies demonstrates that the proposed approach achieves superior performance. In particular, the LSTM model achieved a promising classification accuracy of 99.3%, reflecting its ability to capture key patterns in the beta-thalassemia dataset. To ensure robust performance and prevent overfitting, a five-fold cross-validation strategy was applied, strictly avoiding any data leakage. Its strength lies in modeling relationships among hematological features, such as MCH, RBC, HGB, MCHC, and HCT, which SHAP and LIME consistently identified as highly influential. These findings align with established medical knowledge, confirming that the model relies on clinically meaningful markers. By linking feature importance to pathophysiology, predictions become interpretable and clinically relevant, offering insights beyond accuracy alone. Overall, the results suggest that the LSTM model is both accurate and reliable for supporting beta-thalassemia assessment.

**Table 8:** Comparison of existing studies for beta thalassemia prediction vs. proposed study.

**References**	**Dataset**	**Machine learning**	**Performance metrics**	**XAI**
Ogino et al. (59)	76	LR	Sensitivity **89.2%**, Specificity **92.3%** (initial); Sensitivity **84.4%**, Specificity **88.9%** (validation)	NA
Jahan et al. (50)	3947	ANN, ML on RBC indices	ANN Accuracy **85.95%**	NA
Upadhyay (52)	139	ANN	NA	NA
Kabootarizadeh et al. (51)	268	ANN	Accuracy **92.5%**, Sensitivity **93.13%**, Specificity **92.33%**	NA
(?)	NA	RF, SVM, LR	ML models **94%**, Logistic Regression **95%**	NA
Fu et al. (45)	350	SVM	AUC **0.76**, error rate **0.26**	NA
(?)	NA	Automated microscopy & ML	AUC **0.940**, Sensitivity **84.6%**, Specificity **92.3%**, Severe SCD Sensitivity **97%**	NA
Saleem et al. (55)	10,322	Chi-Square, RFE, LR, GBC	GBC achieved **93.46% accuracy**	NA
Haghpanah (47)	624	RF, GBM, LR	LR: AUC **0.68**, Sensitivity **75%**; RF: AUC **0.68**, Accuracy & Specificity **66%**	NA
Subasinghe et al. (44)	343	SVM, PNN	PNN Model 2 achieved **98.75% accuracy**	NA
Proposed study	5,066	NN, RNN, LSTM	NN Model achieved **94.90% Accuracy** RNN Model achieved **98.00% Accuracy** LSTM Model achieved **99.30% Accuracy**	Yes

## 6 Conclusion

This current research provides an extensive machine learning based technique for utilizing neural networks to identify beta thalassemia quickly and effectively. We developed and compared three frameworks: NN, RNN, and LSTM, using a freely available dataset from Kaggle, where the LSTM model scored the best overall based on all models.

We employed LIME to explain single-patient-level prediction, and SHAP for both local and global feature importance evaluation to significantly enhance model interpretation. These explainability methods enhance the utility of these models in actual healthcare decisions by providing insightful details on how different features contribute to the final predictions.

This research has some limitations, yet it provides favorable findings. The dataset employed, although significant, is limited to samples obtained from a single source from the Punjab Thalassemia Prevention Program from the Punjab province of Pakistan and restricted to a specific region; ethnic/geographical distribution of patients may affect the generality of the findings to the broader population, and prospective or real-world validation is needed. External validation was not possible due to the availability of a single dataset. While high accuracies are observed, rigorous cross-validation and separate testing samples are used to minimize overfitting. The utilization of PCA and SMOT improved the performance of models, but such computational techniques may require conversion for direct clinical application. Additionally, the current approach did not incorporate ensemble learning or hybrid feature selection strategies, which could potentially improve model robustness.

For future work, we plan to extend this research by integrating ensemble learning techniques to combine the strengths of NN, RNN, and LSTM models, and by incorporating advanced feature engineering and optimization algorithms. We also aim to include multi-center datasets from different geographical and racial backgrounds to enhance the universality of the models. Moreover, incorporating clinical domain knowledge and validating the models with real-time hospital data would strengthen their applicability in diagnostic support systems.

## Data Availability

The original contributions presented in the study are included in the article/supplementary material, further inquiries can be directed to the corresponding author.
